# Advancing pathogen surveillance by nanopore sequencing and genotype characterization of Acheta domesticus densovirus in mass-reared house crickets

**DOI:** 10.1038/s41598-024-58768-3

**Published:** 2024-04-12

**Authors:** Fang Shiang Lim, Joel González-Cabrera, Jens Keilwagen, Regina G. Kleespies, Johannes A. Jehle, Jörg T. Wennmann

**Affiliations:** 1 Julius Kühn Institute (JKI) - Federal Research Centre for Cultivated Plants, Institute for Biological Control, Schwabenheimer Str. 101, 69221 Dossenheim, Germany; 2 Department of Genetics and Institute BIOTECMED, Universitat de València, Dr Moliner 50, 46100 Burjassot, Spain; 3 Julius Kühn Institute (JKI) - Federal Research Centre for Cultivated Plants, Institute for the Safety of Biotechnological Processes in Plants, Erwin-Baur-Str. 27, 06484 Quedlinburg, Germany

**Keywords:** Genomic analysis, Next-generation sequencing, Genome assembly algorithms, High-throughput screening, Pathogens, Virology, Metagenomics

## Abstract

Rapid and reliable detection of pathogens is crucial to complement the growing industry of mass-reared insects, in order to safeguard the insect colonies from outbreak of diseases, which may cause significant economic loss. Current diagnostic methods are mainly based on conventional PCR and microscopic examination, requiring prior knowledge of disease symptoms and are limited to identifying known pathogens. Here, we present a rapid nanopore-based metagenomics approach for detecting entomopathogens from the European house cricket (*Acheta domesticus*). In this study, the Acheta domesticus densovirus (AdDV) was detected from diseased individuals using solely Nanopore sequencing. Virus reads and genome assemblies were obtained within twenty-four hours after sequencing. Subsequently, due to the length of the Nanopore reads, it was possible to reconstruct significantly large parts or even the entire AdDV genome to conduct studies for genotype identification. Variant analysis indicated the presence of three AdDV genotypes within the same house cricket population, with association to the vital status of the diseased crickets. This contrast provided compelling evidence for the existence of non-lethal AdDV genotypes. These findings demonstrated nanopore-based metagenomics sequencing as a powerful addition to the diagnostic tool kit for routine pathogen surveillance and diagnosis in the insect rearing industry.

## Introduction

The increased demand for food production and the need to establish food security for the future has led to the emergence of industrial rearing facilities for insects. Industrial scale mass-reared insects, for example the black soldier fly, *Hermetia illucens*^1^, or larvae of the mealworm (*Tenebrio molitor*), can be utilized as an efficient high quality protein source for both, food and feed^2^. In addition to that, mass-reared insects are essential for the application of biological pest control and insect vector control. For instance, mass-reared Lepidopteran larvae are used to produce baculovirus-based biocontrol agents^3^, predators and parasitoids are reared for augmentative biological control^4^ or mass-reared insects are necessary of sterile insect technique (SIT) programms^5^. Beyond these more recent applications, apiculture and sericulture have played an wide cultural and economic role for millennia^6^. Successful application of mass-reared insects, however, relies heavily on maintaining large and healthy insect colonies.

Mass-reared insects are at constant risk of infection by generalist and specialist entomopathogens, including viruses, bacteria and fungi^7^. As an example, the Western honey bee (*Apis mellifera*) is long known to be endangered by bacterium *Melissococcus plutonius,* the infective agent of the European foulbrood (EFB), that can lead to total losses of colonies^8^. In another domain, generalist entomopathogenic fungi from genera of *Beauveria* and *Metarhizium* have been found to infect multiple mass-reared insects, such as *H. illucens* and *T. molitor*^9^. Furthermore, insects may also harbor covert infections that do not produce any visible symptoms. These infections may be maintained unnoticed for several generations, but certain conditions like the stress generated in overcrowded rearing facilities may trigger an overt infection leading to significant loses^9,10^.

As one of the most commonly used insect for food and feed, the European house cricket *Acheta domesticus* has been reared since the eighteenth century with large commercial breeding facilities established both in Europe and North America for the pet industry^11^. While looking into the aspect of entomopathogens, Acheta domesticus densovirus (AdDV) (Family *Parvoviridae*, subfamily *Densovirinae*, genus *Scindoambidensovirus*) is a well-known member of the *Parvoviridae* virus family and infects a broad range of crickets, including *A. domesticus, Gryllus locoroko* and *Gryllus sigillatus*. The virus is rapidly transmitted from insect to insect through fecal–oral route and common symptoms of affected individuals include paralysis, slow growth, poor coordination of movement and high mortality^12^. The first isolation of AdDV was carried out in 1977 and multiple outbreaks of AdDV across Europe, North America and Japan have been reported in rearing facilities for decades^13^. Subsequently, a new volvovirus isolate, Acheta domesticus volvovirus (AdVVV)^14^ (proposed family *Cruciviridae*) was isolated from *A. domesticus* rearing facility in Japan. Furthermore, with the advent of next generation sequencing technologies, Acheta domesticus Iflavirus (AdIV), a novel member of the *Iflaviridae* family, was discovered from frass and insect samples from commercially reared crickets^15^. In the absence of effective surveillance strategies, covert infections are difficult to be detected and eliminated. Conventional molecular methods, such as polymerase chain reaction (PCR)^16,17^ and quantitative PCR (qPCR)^18^, or microscopy analyses have been widely used for the detection of pathogens in insect colonies. However, their application is usually limited to previously known pathogens and it may be difficult to identify the actual pathogen species without more sequencing efforts^19^.

The MinION nanopore sequencing technology is a groundbreaking platform for pathogen identification through genome sequencing (Oxford Nanopore Technologies Oxford, UK). The key innovation of MinION sequencing platform lays in its high portability, which allows sequencing to be done on-site and has potential for real-time data analysis^20^. Several research groups have explored the use of nanopore sequencing for the detection of pathogens and integrated its use in the diagnostics of human, animal or plant diseases^21–25^. Although nanopore sequencing is known for higher error rates than conventional Illumina sequencing, it has demonstrated successful identification of variants of hepatitis C virus^26^ and SARS-CoV^27^ when sufficient sequencing redundancy was achieved. Here, we evaluate the feasibility of utilizing nanopore-based metagenomics sequencing as a rapid strategy for detection of entomopathogens in diseased *A. domesticus*. We have developed a standard post-sequencing bioinformatics pipeline based on Kraken 2^28^ for the detection of entomopathogens in samples of diseased crickets. As part of the portability, the pipeline was integrated into a Nvidia AGX Xavier Developer Kit for bioinformatic analysis. Subsequently, from the downstream sequencing analysis, we obtained metagenome-assembled genomes (MAGs) of AdDV, unraveled the genetic variation and identified different genotypes of AdDV based on nucleotide substitutions.

## Materials and methods

### European house cricket samples

Individuals of *A. domesticus* were provided by a mass-rearing facility producing crickets for commercial use in Italy. This facility has reported that insects showed an increased mortality and typical symptoms of an AdDV infection: lethargy, paralysis, and swollen abdomen. Individuals from three different developmental stages were used in the experiments: (1) nymphs (20 days post hatching), (2) pre-adults (28 to 31 days post hatching) and (3) adults (42 to 50 days post hatching).

Upon arrival, the samples were stored at 25 °C. Within the following days, symptomatic, deceased individuals and crickets without apparent symptoms were sorted and separated into different containers and stored at -20 °C until further use.

### DNA extraction of *A. domesticus*

Twelve symptomatic samples of different life stages were selected. Additionally, six non-symptomatic samples were selected to serve as negative control in the downstream analysis. Total nucleic acids were extracted from entire individual crickets using the ZymoBIOMICS DNA Miniprep Kit (D4300) (Zymo Research Corp., Irvine, CA, USA) according to manufacturer’s protocol with the following modifications. Prior to homogenization, the individual insect was surface-sterilized with sodium hypochlorite solution (0.5%, v/v) for 30 s and subsequently washed in 70% ethanol for 1 min and distilled water for 2 min to remove contaminants. Then, the whole insect was transferred to microcentrifuge tube containing ceramic beads (0.1 and 0.5 mm) and homogenized on a MP Fastprep-24™ (MP Biomedicals, USA) at 6.5 m/s for 30 s, rested on ice for 1 min and homogenized at 6.5 m/s again for 30 s. Prolonged homogenization was avoided to prevent extended DNA fragmentation. DNA was eluted in 75 µL of ZymoBIOMICS™ DNase/RNase Free Water. Quality and quantity of the extracted DNA was assessed by gel electrophoresis in a 1% agarose TAE buffer system and in a Quantus™ Fluorometer (Promega, Winsconsin, WI, USA) using Quanti Fluor® ONE dsDNA system. The extracted DNA was stored at − 80 °C until further investigation.

### Library construction

Library preparation for metagenomics MinION sequencing was achieved using Ligation Sequencing Kit (SQK-LSK109) coupled with Native Barcoding Expansion 1–12 kit (EXP-NBD104) for multiplexing of samples, according to manufacturer’s protocol with slight modifications (Oxford Nanopore Technologies, Oxford, U.K.). In brief, 1 µg of DNA from each sample was end-prepped using NEBNext Ultra II End-repair/dA-tailing module (New England Biolabs, Ipswich, MA). Subsequently, each sample was ligated with an unique barcode (EXP-NBD104) along with NEB Blunt/TA Ligase Master Mix (New England Biolabs, Ipswich, MA) for multiplexing. Equimolar amounts of barcoded DNA from each sample were pooled into a single Eppendorf DNA Lobind Tube (Hamburg, Germany). Lastly, adapter ligation of pooled and barcoded DNA was performed using NEBNext Quick T4 DNA Ligase and NEBNext Quick Ligation Reaction Buffer (New England Biolabs, Ipswich, MA). The eluted library was quantified using a Quantus Fluorometer (Promega, Winsconsin, WI, USA). All clean-up steps during library preparation were conducted using Agencourt AMPure XP beads (Beckman Coulter, Indianapolis, IN) according to manufacturer’s protocol.

### Sequencing and live base calling

The libraries were loaded onto FLO-MIN106D (R9.4.1) flow cells on a MinION Mk1b device (Oxford Nanopore Technologies, Oxford, UK). The device was connected to a NVIDIA Jetson Xavier AGX Developer Kit (NVIDIA, Santa Clara CA, USA) running on Ubuntu (version 18.04.6) as operating system, serving as a control unit for the MinION Mk1b (Oxford Nanopore Technologies, Oxford, UK). Live base-calling using High Accuracy Model (HAC) was performed in Guppy v4.5.3 (https://github.com/topics/guppy) as implemented as part of MinKNOW v4.3.28, in parallel with sequencing.

### Quality filtering and demultiplexing

For bioinformatics analysis, sequences in fastq format were first demultiplexed based on the barcodes used in the library preparation. Sequences generated for each sample were then quality-filtered and trimmed to keep sequences with quality score greater than seven (Q > 7) and length longer than 100 bases using NanoFilt v2.7.1 (https://github.com/wdecoster/nanofilt). Read statistics for each sequencing run were obtained using NanoStat v1.5.0 (https://github.com/wdecoster/nanostat), implemented as part of the NanoPack package^29^.

### Host removal and taxonomic assignment

Quality filtered reads were first aligned to the genome of *A. domesticus* (GCA_014858955.1, NU_Adom_1.1) using minimap2 (https://github.com/lh3/minimap2) with the option for noisy long reads (map-ont). Mapped reads, which most likely belong to the host were removed for the subsequent analysis using samtools (https://github.com/samtools/samtools). The taxa composition of unmapped reads were assigned using Kraken 2 v2.1.1^28^ against an in house custom-built index (RFGINV_JKI_ID 1.0). This index consisted of reference nucleotide sequences of the domains Bacteria and Archaea, kingdom Fungi, superkingdom of Viruses and Invertebrates (https://ftp.ncbi.nlm.nih.gov/genomes/refseq/) and retrieved from the National Centre for Biotechnology Information Reference Sequence Database (NCBI RefSeq) (The library, created on 27 December 2020, can be inspected in the supplementary file (S1. Appendix)). The minimum confidence score threshold for taxonomy assignment was set to 0.05 to remove false positive assignments. Kraken 2 output was imported into the Pavian interactive web application (https://github.com/fbreitwieser/pavian) to visualize the respective taxa present in each sample^30^.

### De novo AdDV genome assembly

After taxonomic classification, sequence reads assigned to AdDV (NCBI taxonomy entry name: *Orthopteran scindoambidensovirus 1*; NCBI taxonomy ID: 2745121; Naming according to ICTV: *Scindoambidensovirus orthopteran1* (accessed on 7th November 2023)) were extracted from the output of Kraken 2 using KrakenTools v1.2 (https://github.com/jenniferlu717/KrakenTools; extract_kraken_reads.py script). For each of the sequenced samples, a de novo genome assembly was performed using the extracted reads. MAGs of AdDV were generated from the MinION reads using Canu v2.2^31^ with default parameters for ONT sequencing (-nanopore-raw), and an estimated genome size of 6 kb using the extracted sequence reads for each sample. Extracted reads were mapped back to the draft genome using minimap2 v2.17 with map-ont parameter and sorted using Samtools v1.9^32^. Coverage of the assembly was calculated using Qualimap v2.2.1^33^. Draft genomes generated by Canu were polished using Medaka v1.4.4 (https://github.com/nanoporetech/medaka) with default parameter. For the de novo assembled and polished AdDV genomes, CheckV v1.0^34^ was used to assess their completeness and quality.

### Phylogenetic analysis

For phylogenetic analysis, the de novo assembled genomes were aligned using MAFFT v7 webserver (https://mafft.cbrc.jp/alignment/server/)^35^ together with other AdDV sequences publicly available at NCBI Genbank (S2 Table), previously described from infection outbreaks over the past decades. Multiple sequence alignment was subjected to gblock trimming to remove poorly aligned regions and preserve conserved regions^36,37^. Maximum likelihood (ML) phylogenetic reconstruction was performed on the trimmed and aligned whole genome nucleotide sequences. The analysis was performed using IQ-Tree v1.6.12^38^, following BICs selection of the best substitution model TPM3 + F + G4, implemented in ModelFinder^39^ and 1000 ultrafast bootstrap approximation for assessing node support. The phylogenetic tree was visualized with iTOL^40^.

### De novo variant calling analysis for AdDV

The detection of single nucleotide variations (SNVs) for each assembled AdDV genome was performed as follows: (1) For each sequenced sample, the reads that were previously assigned belonging to the AdDV (see Sect. 2.7) were mapped to reference sequence AdDV (NC_004190.1) with minimap2, resulting in twelve binary alignment files (BAM). (2) SNV positions were called using neural network-based callers Medaka v1.7.2 to determine respective SNV positions across all sequenced samples. Based on the common reference genome used for the SNV determination, 100 variable SNV positions were detected shared among all twelve samples. (3) The occurrences of nucleotide frequencies in these positions were counted using MPileup v1.16.1. For each sample, the nucleotide frequencies were plotted in R (R v4.2.1 in RStudio v2022.07.1 + 554).

### AdDV genotype determination

To link each SNV positions on each read, the BAM files from the de novo variant calling were used to determine the nucleotides occurring on each read in the 100 variable positions using pileup software. Only reads with length between 1000 and 6000 bp and covering at least 10 SNVs were considered for linkage and genotyping analysis. A maximum of 1000 longest reads were used for subsequent analysis, whenever it was possible.

For each sample, this resulted in a matrix where each row was represented by a read and the columns indicated the nucleotides occurring in each position. This nucleotide matrix was used to calculate a dissimilarity (*D*) matrix using the following function:

$$D = \frac{{\left( {0.5 \times No. \;all\; SNV\;positions} \right) + No. \;non \;shared \;nucleotides}}{No. \;all\;SNV\;positions + No. \;shared\; nucleotides}$$

Two reads that have no overlap (= do not cover a common position) receive a dissimilarity of *D* = 0.5. A dissimilarity of *D* < 0.5 indicates an overlap with identical nucleotides. A dissimilarity of D > 0.5 means that the overlap of two reads is based on non-identical nucleotides (mismatches). The greater the overlap with identical or different nucleotides, the smaller or larger the value of *D*, respectively. The obtained distance matrix was used to determine clusters using hierarchical clustering in R. Single linkage was chosen as the clustering method, whereby reads were assigned to each other according to the smallest distance. Reads that formed a continuous clade in the single linkage clustering were extracted and used for de novo genome assembly using Canu v2.2^31^.

### Hierarchical clustering on principal components (HCPC)

The variability of AdDV isolates from each sample, differentiated by SNV positions and frequencies can be assessed by the distances between individuals using factorial analysis (PCA), HC and k-means clustering, forming HCPC. The SNV frequency table consisting of 100 SNV positions was used by applying the HCPC method as implemented in the FactoMineR v2.8 package^41,42^ in R (R v4.2.1 in RStudio v2022.07.1 + 554). Hierarchical cluster tree and factor map were generated, representing the homogeneous and mixture of AdDV isolates within the same colony.

## Results

### Nanopore sequencing

**Figure 1:**
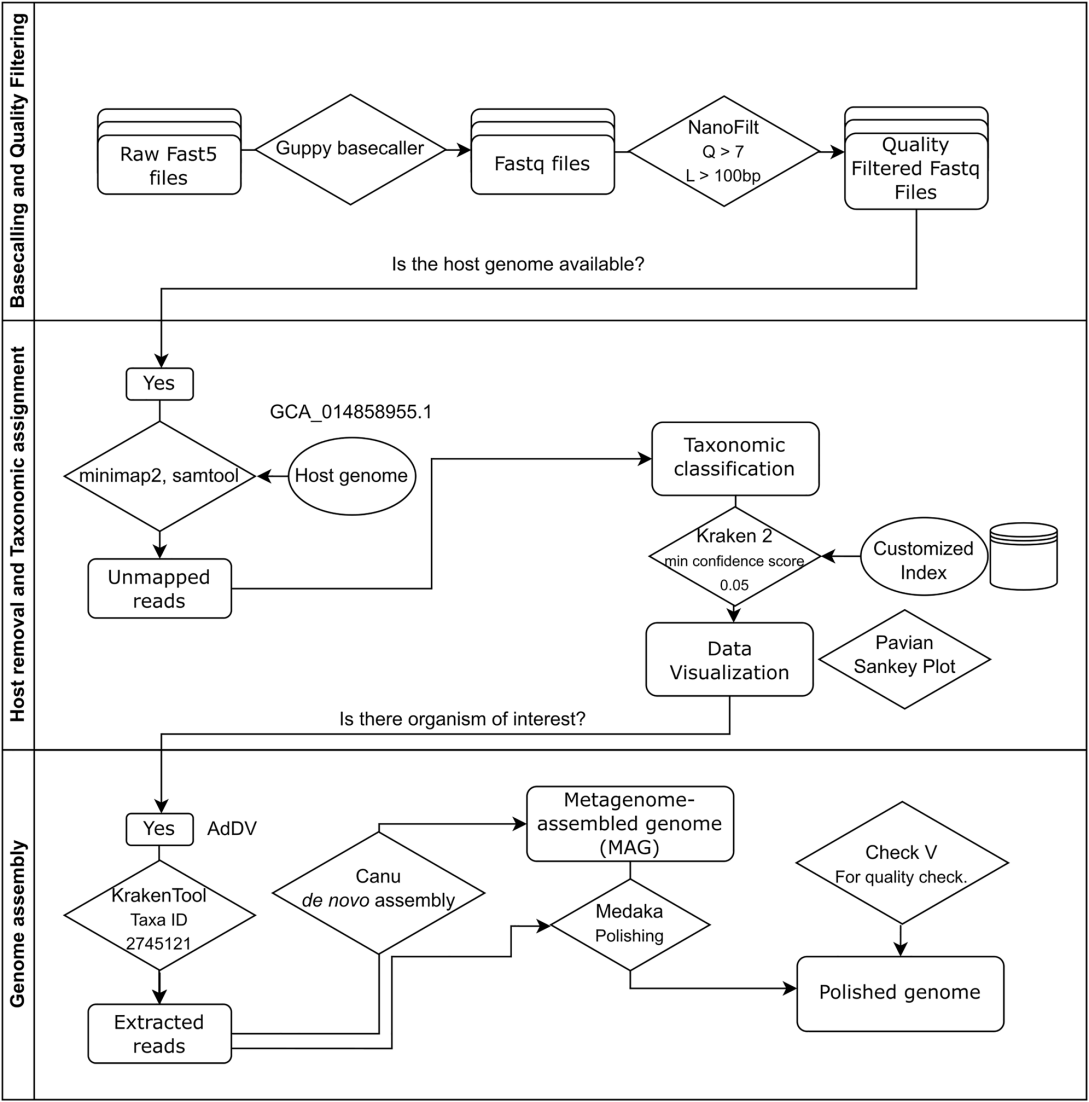
Schematic diagram of the bioinformatics workflow for entomopathogen detection based on nanopore sequencing metagenomics analysis.

To detect the presence of any potential entomopathogen within the diseased house crickets, twelve symptomatic samples, as well as six non-symptomatic samples were collected and subjected to a metagenomics workflow (Fig. 1). For individuals received alive, it was easy to determine the life stages. However, this was very difficult for individuals received dead since melanisation, missing limbs and/or decomposition of the sample had occurred. Total DNA was extracted from individual insects and sequenced, resulting in sequences of the host, as well as microbes and viruses. The sequencing was performed in three separate sequencing runs, each run comprising six barcoded samples. Run #1 included DNA extracted from symptomatic alive nymphs, pre-adults and adults, run #2 was performed on the DNA extracted from deceased cricket samples whereas run #3 included DNA extracted from non-symptomatic samples. The sequencing runs were performed for 18 to 20 h in the same flow cell. After quality filtering of reads, average number of quality reads generated for each individual ranged from 0.2 to 1.2 million reads corresponding to 3.73 × 10^8^ to 1.65 × 10^9^ sequenced bases. The mean quality of the filtered reads ranged from 12.1 to 13.7. The overall read length differed between the three sequencing runs: In run #1, the average read length and N50 ranged between 455 and 1310 bp, run #2 with the deceased samples yielded a considerable higher N50 of 2261 to 5276 bp and run #3 with the non-symptomatic sample yielded N50 of 4820 to 8361 bp (Table 1).

**Table 1:** Sequencing summary for all *A. domesticus* samples, both symptomatic and non-symptomatic that were subjected to a metagenomics workflow for the detection of entomopathogens.

Run	Sample	Vital status	Symptomatic^a^?	Life stage	Number of reads	Mean read length	Mean read quality	N50	Total bp	Reads < Q7 (%)
#1	S1	Alive	Yes	Adult	1,158,216	386.3	13.2	455	4.47 × 10^8^	2.4
	S2	Alive	Yes	Adult	706,623	756.6	13.6	1137	5.34 × 10^8^	2.5
	S3	Alive	Yes	Preadult	388,837	962.7	12.4	1310	3.73 × 10^8^	3.2
	S4	Alive	Yes	Preadult	675,649	704.3	13.3	959	4.75 × 10^8^	2.6
	S5	Alive	Yes	Nymphs	841,766	794.1	13.2	1088	6.68 × 10^8^	2.4
	S6	Alive	Yes	Nymphs	605,435	762.4	13.2	1030	4.61 × 10^8^	2.3
#2	S7	Dead	Yes	n.i*	565,642	2366	13.7	3815	1.33 × 10^9^	1.8
	S8	Dead	Yes	n.i	287,947	2.671	13.5	5276	7.69 × 10^8^	3.4
	S9	Dead	Yes	n.i	495,041	1272	13.8	2261	6.29 × 10^8^	2.1
	S10	Dead	Yes	n.i	604,034	1804	13.7	2874	1.09 × 10^9^	3.1
	S11	Dead	Yes	n.i	481,309	3.015	13.7	4396	1.45 × 10^9^	3.6
	S12	Dead	Yes	n.i	389,788	2975	13.7	5080	1.15 × 10^9^	3.2
#3	S13	Alive	No	Adult	300,120	4977.7	12.3	7116	1.50 × 10^9^	3.1
	S14	Alive	No	Adult	309,130	5327.9	12.2	7159	1.65 × 10^9^	4.6
	S15	Alive	No	Preadult	194,522	5590.6	12.1	8362	1.09 × 10^9^	3.4
	S16	Alive	No	Preadult	277,420	5420.2	12.5	7565	1.50 × 10^9^	4.1
	S17	Alive	No	Nymphs	294,555	4834.3	12.1	7065	1.42 × 10^9^	2.4
	S18	Alive	No	Nymphs	291,518	4830.9	12.1	6976	1.41 × 10^9^	3.9

*n.i, not identified.

*a, symptoms of lethargy, paralysis, and swollen abdomen observed.

The host genomic information was removed from the samples, by mapping to the host reference genome, leaving only the unmapped reads of interest. Approximately 43,513 (22%) to 586,304 (51%) of the total sequencing reads remained for each sample after initial *A. domesticus* host read removal. To identify potential disease causing microbial and viral pathogens in the samples, these reads were taxonomically assigned to the RFGINV_JKI_ID 1.0 index. After taxonomic classification, we obtained a range of 8.7% to 69.3% classified reads across the 18 sequenced samples. A significant number of unclassified reads was observed in several samples, including S1, where about 89% (corresponding to about 535,000 reads) could not be classified as any taxon. To ensure we are not missing out on any potential pathogens, unclassified reads were extracted from respective sample and de novo assembly was performed. The assembled contigs were subjected to BLASTn analysis against the non-redundant nucleotides database (nt). Based on the BLAST analysis, the majority of the contigs were assigned to either *A. domesticus* complete mitochondrion genome with 100% query coverage, or to a closely related insect species (*Gryllus bimaculatus*, *Gryllus veletis*) and other Eukaryotes with relatively lower query coverage (Full table of best hit can be found in supplementary file S3 Appendix). Other matches included newly described bacteria, such as *Entomomonas* sp. F2A isolated from the *A. domesticus*^43^.

There was a range of 5.1% to 97% of classified reads assigned to putative invertebrates. The classified reads for each sample matching to the domain of Bacteria ranged from 0.5 to 58.5% of total classified reads. On the other hand, reads assigned to viruses ranged from 0.4 to 80.2% (Table 2). Approximately 0.7% to 6.1% of all reads were assigned ambiguously to Bacteria, Fungi and Viruses, but were not considered as unclassified. This is a prevalent issue in Kraken 2 analysis because it exclusively assigns reads based on a confidence score from unique regions based on k-mers^44^.

**Table 2:** Sequencing summary of samples of the *A. domesticus*.

Sample	Total reads^a^	Non host reads^b^	Unclassified reads	Classified reads	Putative invertebrates^c^		Bacteria		Viruses		Not matching filter criteria^d^	
					Reads	(%)	Reads	(%)	Reads	(%)	Reads	(%)
S1	1,158,216	586,304	535,474	50,830	45,490	89.5	1528	3.0	717	1.4	3095	6.1
S2	706,623	371,024	290,097	80,927	75,452	93.2	1141	1.4	2,578	3.2	1756	2.2
S3	388,837	184,067	155,328	28,739	25,828	89.9	1934	6.7	212	0.7	765	2.7
S4	675,639	458,462	200,362	258,100	47,844	18.5	1262	0.5	204,357	79.2	4637	1.8
S5	841,766	425,690	340,202	85,488	82,958	97.0	478	0.6	391	0.5	1661	1.9
S6	605,435	296,656	248,889	47,767	45,908	96.1	334	0.7	301	0.6	1224	2.6
S7	565,642	237,611	146,017	91,594	19,350	21.1	52,431	57.2	19,127	20.9	686	0.7
S8	287,947	220,713	67,672	153,041	7737	5.1	89,237	58.3	54,995	35.9	1072	0.7
S9	495,041	208,008	180,262	27,746	26,581	95.8	349	1.3	119	0.4	697	2.5
S10	604,034	216,274	171,615	44,659	27,780	62.2	824	1.8	15,289	34.2	766	1.7
S11	481,309	145,983	103,839	42,144	17,842	42.3	4,183	9.9	19,493	46.3	626	1.5
S12	389,787	130,201	94,162	36,039	14,845	41.2	3,097	8.6	17,576	48.8	521	1.4
S13	300,120	111,968	41,937	70,031	66,855	95.5	438	0.6	1156	1.7	1582	2.2
S14	309,130	63,326	49,156	14,170	13,512	95.4	395	2.8	50	0.4	213	1.5
S15	194,522	43,211	33,865	9346	8778	93.9	433	4.6	16	0.2	119	1.3
S16	277,420	149,213	28,119	121,094	115,738	95.6	496	0.4	2111	1.7	2749	2.3
S17	294,555	92,573	49,928	42,645	41,059	96.3	1242	2.9	104	0.2	240	0.6
S18	291,518	87,930	48,360	39,570	38,260	96.7	1027	2.6	60	0.2	223	0.6

The total number of reads reflects the raw reads that were assigned by barcode to this sample. Classification was done with Kraken 2 starting from the domain level. Non host reads represent the unmapped reads post mapping to *A. domesticus* genome (GCA_014858955.1, NU_Adom_1.1).

^a^Raw reads that were assigned by barcoding to this sample.

^b^Unmapped reads post mapping to *A. domesticus* genome (GCA_014858955.1, NU_Adom_1.1).

^c^Reads that were assigned to genomes of domain Eukaryota that are present in the index.

^d^Reads not going down to domain level.

**Figure 2:**
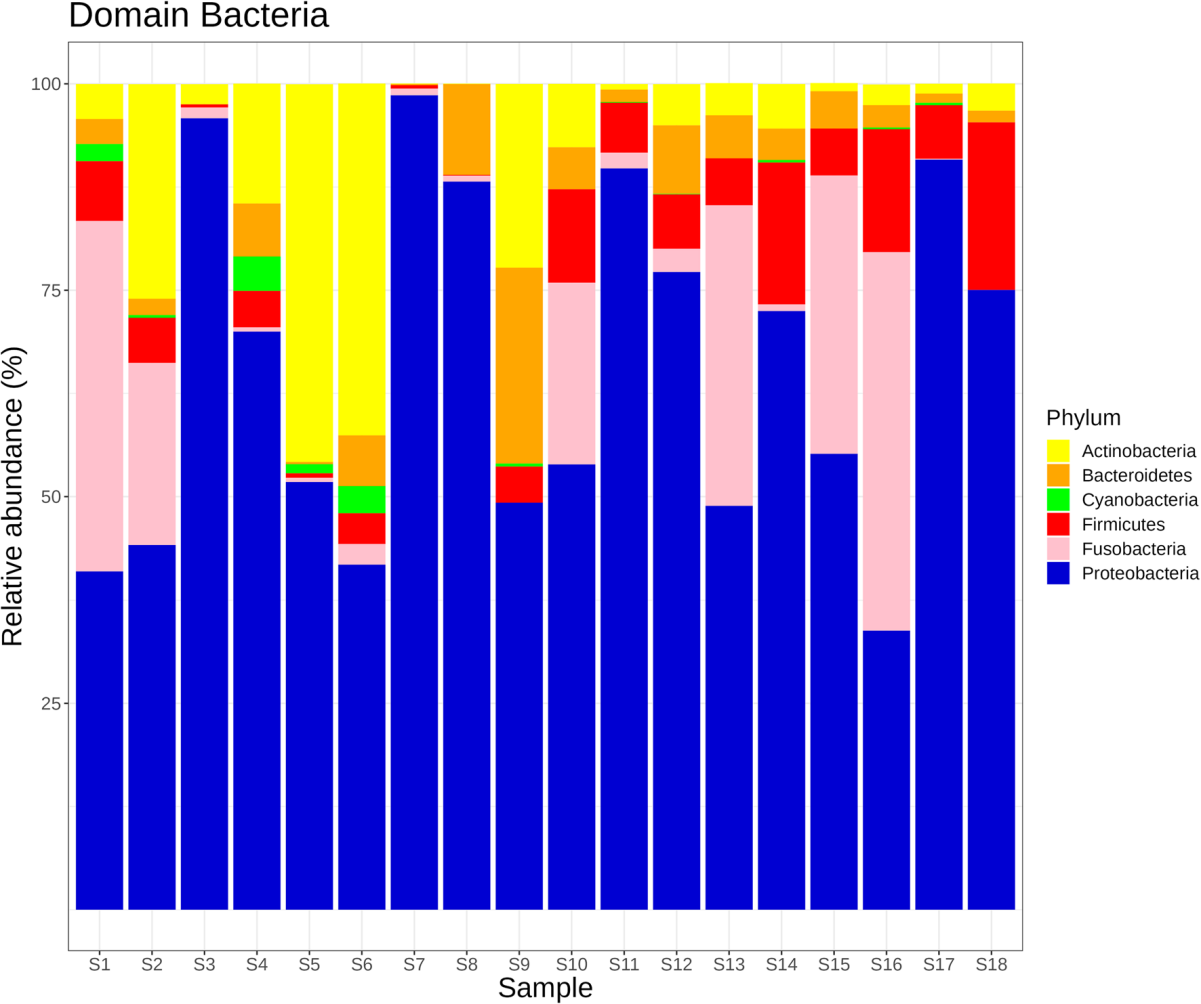
Relative abundance (%) of bacterial reads for 18 sequenced samples of *A. domesticus* at phylum level based on metagenomics analysis using in house custom-built index (RFGINV_JKI_ID 1.0). The figure displays the six most abundant bacteria phyla assigned. Relative abundance was calculated by dividing reads assigned to specific phyla to total number of bacterial reads.

Within Bacteria, reads were mainly dominated by bacteria of the phylum Proteobacteria, with up to 98.6% (S7) of total bacterial reads (Fig. 2). The next two most abundant bacteria belonged to the phyla Actinobacteria, attributing up to 45% of the total bacterial reads in S5 and Bacteroidetes, contributing to 20% of total bacteria reads in S9. The relative abundance of the bacterial phylum Fusobacteria was found to be higher in S1 (42.4%), S2 (22.0%), S10 (22.0%), S13 (36.4%), S15 (33.7%) and S16 (45.8%) compared to other samples. Other bacterial phyla were Cyanobacteria and Firmicutes. Within the Proteobacteria, the majority of reads was assigned to the genus of *Citrobacter*, followed by *Enterobacter*, *Acinetobacter*, and *Klebsiella*. While in the phylum of Fusobacteria, the reads mostly belonged to the genus of *Fusobacterium*. Sankey plot for each sample, together with the full taxonomic assignment list generated from Kraken 2 output can be found in the Supplementary file S4 Figure and S5 Appendix.

We investigated further into those reads that were initially categorized belonging to the category Viruses (as indicated in Table 2), ranging from 119 reads (sample S9) to 200,000 reads (sample S4), to species level. In all symptomatic samples, except for S9, high relative abundance of AdDV was found, with proportions of AdDV exceeding 99.8% (Table 3). In sample S9, 92 of the 119 (77.3%) viral reads were classified as AdDV, whereas 27 virus reads (22%) were assigned to the Invertebrate iridescent virus 6 (IIV6). In comparison to symptomatic samples, non-symptomatic samples exhibited a relatively low numbers of AdDV reads (S14 to S16 = 0; S18 = 1; S17 = 3 and S13 = 9) but with higher relative frequencies of reads assigned to IIV6 for sample S13 (97.8%) and S16 (99.5%).

**Table 3:** Reads assigned to Acheta domesticus densovirus (AdDV) and Invertebrate iridescent virus 6 (IIV6).

Sample	Reads assigned to viruses	Reads assigned to AdDV and proportion to viruses (%)		Reads assigned to IIV6 and proportion to viruses (%)*	
S1	717	711	(99.2)	n.a	(0)
S2	2578	2576	(99.9)	n.a	(0)
S3	212	212	(100)	n.a	(0)
S4	204,357	204,346	(99.9)	n.a	(0)
S5	391	391	(100)	n.a	(0)
S6	301	301	(100)	n.a	(0)
S7	19,127	19,126	(99.9)	n.a	(0)
S8	54,995	54,992	(99.9)	n.a	(0)
S9	119	92	(77.3)	27	(22)
S10	15,289	15,257	(99.8)	n.a	(0)
S11	19,493	19,491	(99.9)	n.a	(0)
S12	17,576	17,573	(99.9)	n.a	(0)
S13	1156	9	(0.007)	1130	(97.8)
S14	50	n.a	(0)	n.a	(0)
S15	16	n.a	(0)	n.a	(0)
S16	2111	n.a	(0)	2100	(99.5)
S17	104	3	(2.9)	1	(0.01)
S18	60	1	(1.7)	5	(8.3)

*n.a., non assigned.

Reads assigned to AdDV were extracted and used for de novo whole genome assembly for each sample independently. We were not able to construct any assembly out of non-symptomatic samples due to the low number of AdDV reads that did not cover the entire length of the AdDV genome. For all twelve sequenced symptomatic samples, the reconstruction of the entire AdDV genome was successful leading to contigs from 5.5 to 6.1 kb. BLAST analysis revealed that these genomes shared 98.9% to 99.7% identity to other known AdDV isolates, including AdDV isolate AdJP12 isolated from Japan in 2012 and AdDV isolate AdNA09 isolated from North America in 2009^45^. Based on CheckV viral genome evaluation, the polished genomes were estimated to be of high quality. In general, all five ORFs, non-structural (NS) 1, 2, 3 and structural (VP) 1 and 2 genes were found in all MAGs. Terminal repeats were only determined in S4, S7 and S10. The MAGs from this study were uploaded to NCBI (Accession number PP054196 to PP054217).

**Figure 3:**
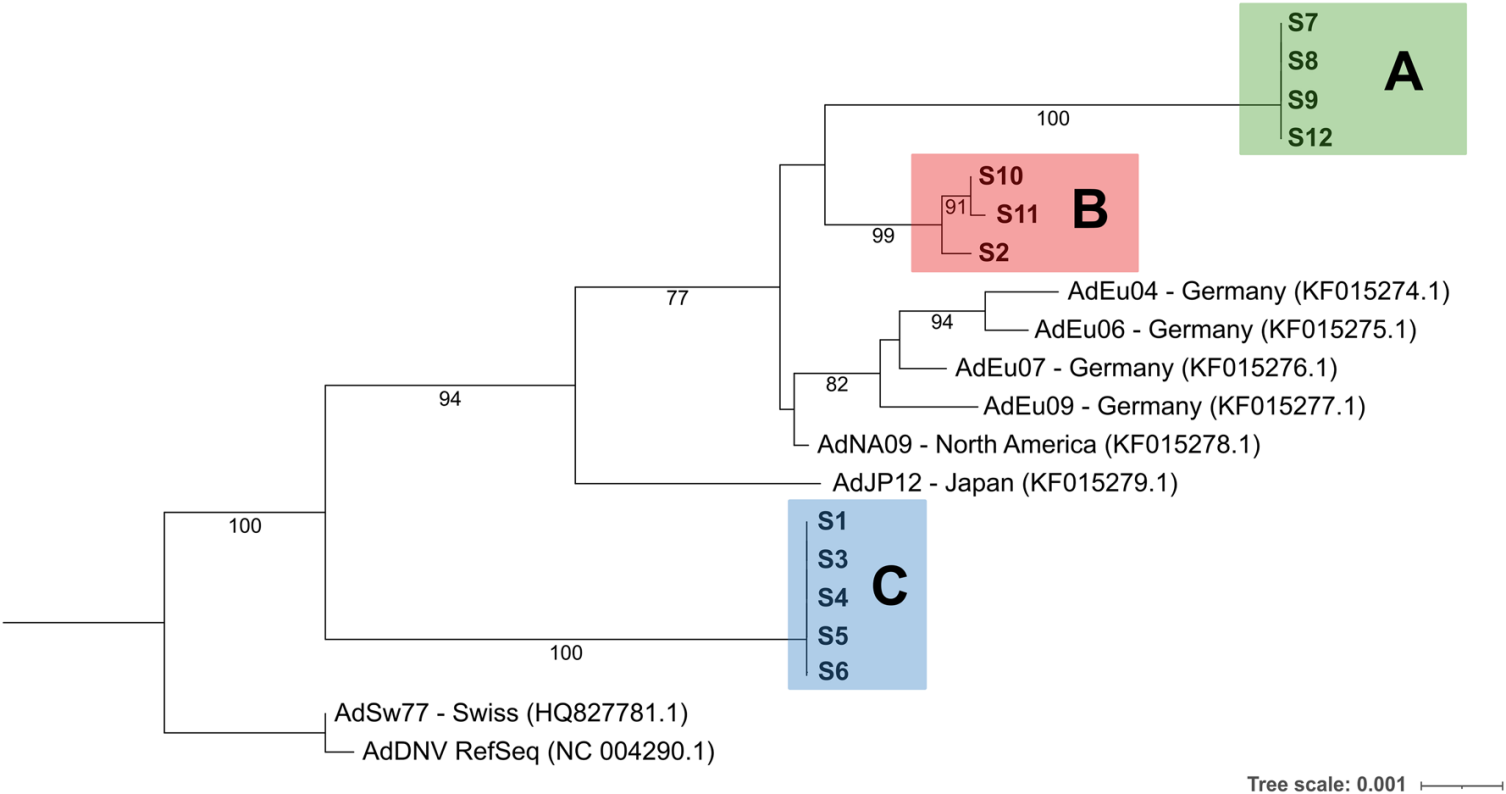
Maximum-likelihood phylogeny (5233 positions) based on alignment of AdDV whole genome nucleotide consensus sequences, using best-fit model according to BIC. Value on each node representing bootstrap value (1000 replicates). Only values of > 70% are displayed in the tree. The newly assembled genomes from this study are shown in bold and fall into three clades A (green), B (red) and C (blue).

The trimmed alignment comprised 5233 nucleotide positions, including the complete ORFs of the AdDV genome. Based on this alignment, the phylogenetic relationship of the twelve newly assembled AdDV genomes and eight previously published AdDV sequences was reconstructed (Fig. 3). Here, the twelve AdDV of this study separated into three distinct clades: A (S7, S8, S9 and S12), B (S2, S10 and S11) and C (S1, S3, S4, S5 and S6). Clade A and B were sister clades and both subclades of AdDV reported from Germany, North America and Japan (Fig. 3). Clade C was more basal than clade A and B and closer related to the isolates from Switzerland^46^ and AdDV reference sequence from NCBI RefSeq. Notably, clade A and B comprised the AdDV sequences of deceased *A. domesticus* samples, except for sample S2. Clade C comprised the AdDV sequences that were extracted from alive but symptomatic *A. domesticus* samples (S1, S3, S4, S5 and S6) (Table 1, Fig. 3) and was more closely related to isolate AdSw77 (HQ82778.1).

**Figure 4:**
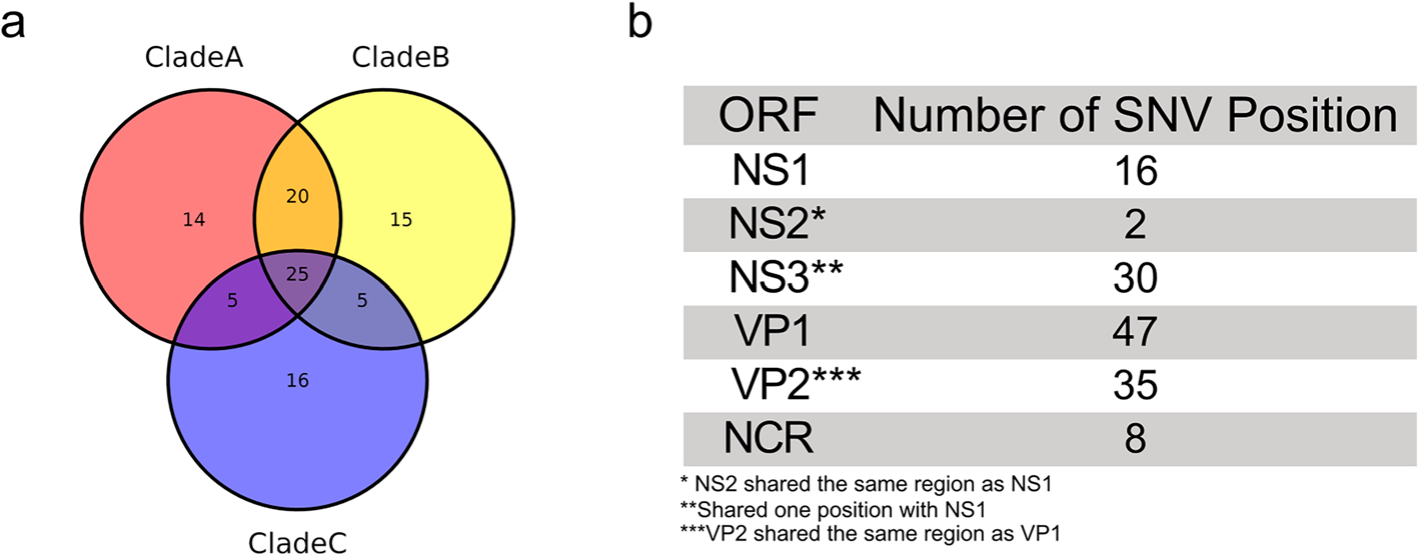
Distribution of single nucleotide variation (SNVs) within (**a**) clade A, B and C and (**b**) deriving from the ML phylogeny and their respective positions in the genome. Clade A, B and C have a total number of 14, 15 and 16 SNV positions, which were unique to their respective clade.

To analyze the intra-species genetic diversity of the twelve sequenced AdDV samples, variable SNV positions were detected after mapping of the extracted AdDV reads to a common reference genome AdDV (NC_004290.1) obtained from NCBI RefSeq database. An isolate-spanning nucleotide substitution analysis allowed linking detected SNV positions among isolates to the common reference sequence. Using this method, a total of 100 SNV positions was identified in all twelve analyzed datasets. Twenty-five SNV positions were shared between all twelve AdDV samples, whereas the remaining 75 SNV positions were either unique or shared by less than twelve sequenced samples. For clades A, B and C, a total number of 14, 15 and 16 positions were unique, respectively. Among the 100 SNV positions, 92 and 8 SNV positions were located in coding region and non-coding regions, respectively (Fig. 4a). Overall, the 92 SNV positions were unevenly distributed between the five ORFs (NS1, NS2 and NS3, VP1 and VP2) of the AdDV genome. Out of the 92 SNV positions in coding regions, 45 were found in NS genes and 47 SNVs belonged to VP genes. Most SNV positions were found in VP1 (47 SNV positions) and NS3 (30 SNV positions), respectively (Fig. 4b). Since VP1 and NS3 are 2451 bases and 642 bases long, the SNV density was higher in NS3 than VP1. Only 2 SNV positions were found in NS2, which is located within NS1, with only 16 variable SNV positions (Fig. 4b). In addition, on top of the SNVs’ distribution across the genome, we also analysed their impact on the amino acid sequences (synonymous vs non-synonymous substitutions). When non-synonymous SNVs were taken into account, NS3 was found to carry 26 SNV positions while in VP1, 20 out of 47 SNV positions were found to have an effect on the encoded protein.

### Phylogenetic analyses

**Figure 5:**
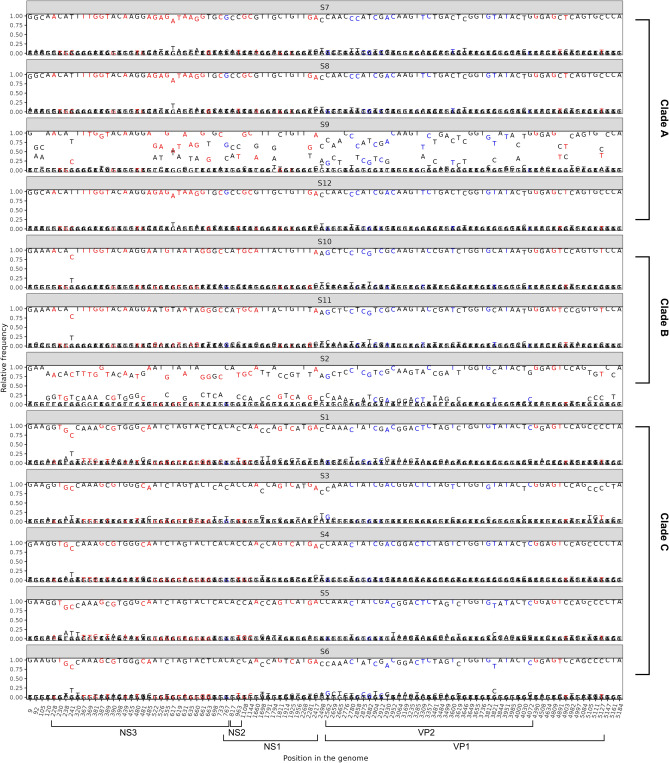
Nucleotide frequency of identified SNV positions for all samples from each clade, A (S7, S8, S9 and S12); B (S10, S11 and S2); C (S1, S3, S4, S5 and S6). Predominantly 1.0 and 0 of relative nucleotide frequency was observed, suggesting homogenous AdDV genotype for the samples S7, S8, S12, S10, S11, S1, S3, S4, S5 and S6. Sample S9 (Clade A) and sample S2 (Clade C) have distinct SNV frequencies of 0.4 to 0.6 and 0.2 to 0.8, respectively, suggesting a mixture of AdDV genotypes. Nucleotide in red indicates non-synonymous substitution in one coding sequence; blue indicates non-synonymous substitution in two coding sequences and black indicates synonymous substitution.

Phylogenetic analyses that are based on de novo assembled consensus sequences neglect possible genetic variations that occur within a virus population, as found in the individual sequenced samples. Hence, the twelve samples were checked for intra-sample genetic diversity by calculating the frequencies of the four nucleotides (A, T, G and C) in each of the 100 detected SNV positions (Fig. 5). In case of a genetically homogeneous AdDV sample, the nucleotide frequency at each SNV position was expected to be at either close or equal to 0 or 100%, as it was the case for S1, S3, S4, S5, S6, S7, S8, S10, S11 and S12 (Fig. 5). In case of a sample with potential mixture of genotypes, the frequencies were expected to aggregate at a level between 0 and 100%, which was detected for S2 and S9 (Fig. 5). According to the SNV frequency analysis, all samples from the phylogenetic clade C, which consisted only of symptomatic alive *A. domesticus* samples, were genetically homogenous, whereas in clade A and B, all but samples AdDV S2 and S9 were found to show a homogenous pattern (Fig. 5).

**Figure 6:**
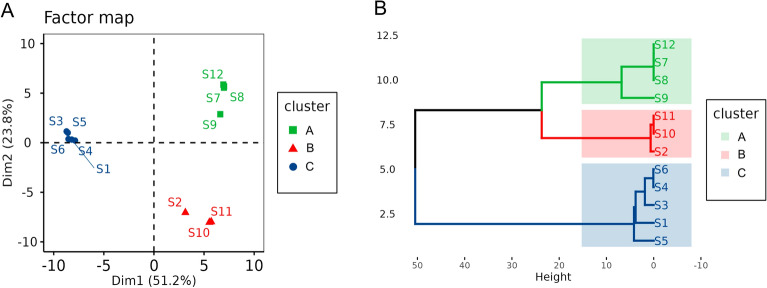
HCPC analysis of twelve AdDV samples S1 to S12 based on identified SNVs data set. (**A**) Relative position of samples AdDV S1 to S12 in a two-dimensional factor map evaluated by principal component analysis. Each point representing a sample and different clusters were distinguished by colors. (**B**) Hierarchical tree suggests a clustering into three clusters. Cluster A, B and C were generated, as indicated by the different colours. The factor map and hierarchical cluster were generated using FactoMinerR v2.8 package in R.

Closer examination on the pattern of alternative nucleotides in AdDV from sample S2 suggested that this sample is a mixture of an AdDV belonging to clade B and clade C in a ratio of about 0.8 to 0.2 (Fig. 5). Similarly, AdDV from sample S9, appeared to contain 60% clade A and 40% clade B specific SNVs (Fig. 5). To inspect the genetic variation and similarity among the twelve samples, HCPC analysis based on the SNV frequency and position was performed (Fig. 6). The genetic composition of the twelve samples represented by their SNVs were reflected by their relative positions in the HCPC plot. Again, all twelve samples were grouped into three major clusters (A, B, C) which correspond to the phylogenetic clades A, B, C, respectively (compare Fig. 3). In the HCPC factor map, sample S9 of cluster A appears to be slightly shifted towards cluster B, while sample S2 of cluster B is shifted towards cluster C, underlining their mixed nucleotide composition of cluster A and B, and cluster B and C, respectively (Fig. 6A).

### Genotype resolution

Since nanopore reads were possibly long enough to cover entire AdDV genomes, a SNV-based approach was followed to determine the genotypic composition of the twelve nanopore sequenced AdDV samples. For this approach the number of reads was reduced to the longest reads of each sample that covered at least 10 of the 100 previously detected SNV positions. The idea was that for each read and covered SNV position the occurring nucleotide was noted and summarized in a SNV nucleotide matrix. From this nucleotide matrix, the distance was calculated and was used for hierarchical clustering using single linkage algorithm.

**Figure 7:**
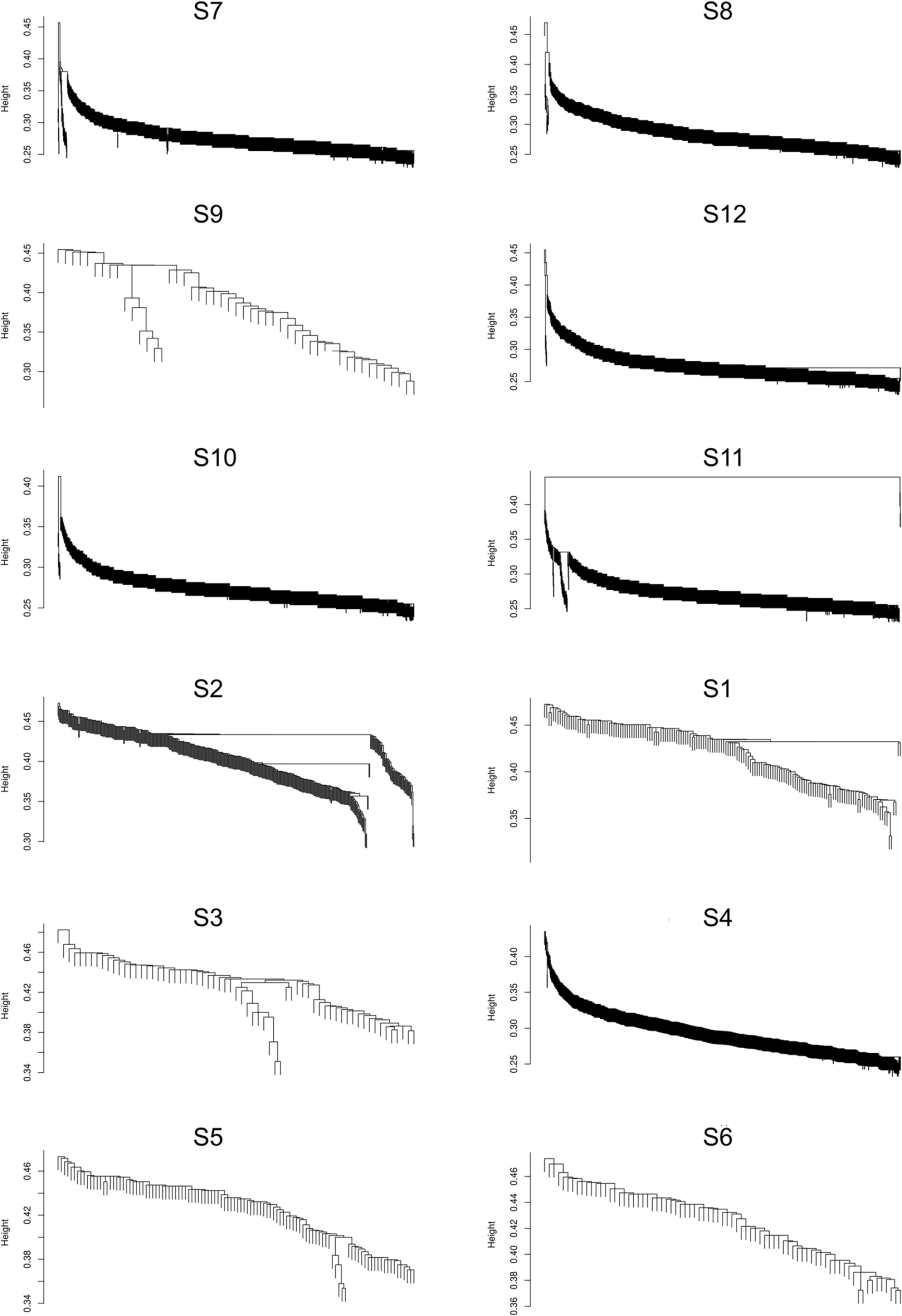
Single linkage cluster dendrogram for all samples from each clade, A (S7, S8, S9 and S12); B (S10, S11 and S2); C (S1, S3, S4, S5 and S6). Only reads that are longer than 1000 bp, covering at least 10 SNV positions were used in the analysis. Dendrogram was plotted using ggplot2 v3.5.0 package in R based on maximum of the 1000 longest read for samples S4, S7, S8, S10, S11 and S12. Only 160, 65, 110, 77 and 49 reads were used for sample S1, S3, S5, S6 and S9, respectively.

Typically for clustering by single linkage, a chain-like arrangement of reads was observed for each sample. For samples that were assumed to be homogenous, a long continuous single arrangement of reads is expected, as observed in the samples S1, S4, S5 and S6 (Fig. 7). The samples AdDV S2 and S9 were splitted into two main branches, representing the two genotypes present in the mixtures (Fig. 7). Based on the SNV nucleotide frequency plots, it was assumed that all twelve samples, except S2 and S9, were genetically homogeneous. However, despite the observation of a clear continuous chain of reads in the remaining samples, small split-offs were visible, indicating heterogeneities in sample S3, S7, S8, S10, S11 and S12 (Fig. 7).

**Figure 8:**
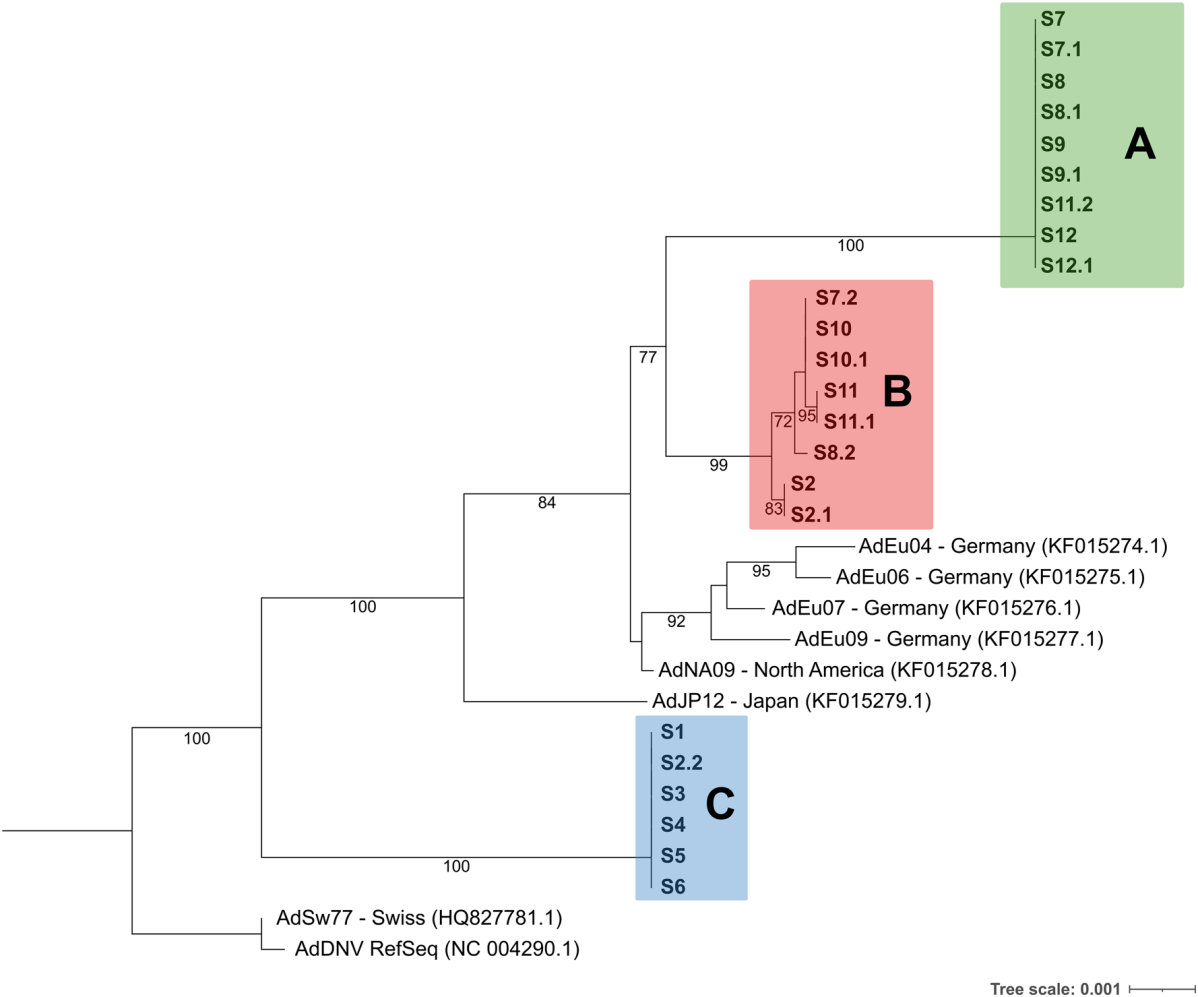
Maximum-likelihood phylogeny (from 5201 genome positions covering 100 SNV positions) based on alignment of AdDV whole genome nucleotide consensus sequences, using best-fit model according to BIC. The value on each node represents the bootstrap value of 1000 replicates. Only values of > 70% were displayed in the tree. SX.1 representing the major genotype and SX.2 representing minor genotype, each  separated from the mixture of genotypes.

To check whether these findings derived from sequencing errors or from potential sub-genotype sequences in the respective samples, reads were extracted from the major and minor branches and subjected subsequently to a de novo assembly. The construction of two MAGs was successful for samples S2, S7, S8 and S11. For samples S3, S9, S10 and S12, we were not able to reconstruct the MAG from the minor branch due to the small number of reads available. To examine which clade the newly constructed MAGs from the different samples belonged to, phylogenetic analysis was performed, together with the original AdDV sequences, as well as the newly constructed MAGs genotypes (Fig. 8).

In line with Fig. 3, most of the phylogenetic relationships of AdDV isolates remained unchanged, and three distinct clades A to C were recognized for the samples of this study. Notably, sample S2 exhibited a separation of the mixture into a major genotype S2.1 in clade B and a minor genotype S2.2 in clade C, which reflected the result of the nucleotide frequency plot (Fig. 6A). For AdDV samples S7, S8 and S11 additional minor MAGs were reconstructed by the de novo assembly of the minor branches, consisting of only 21, 11 and 22 reads, respectively. Since these few reads corresponded to only 0.02 to 0.1% of all S7, S8 and S11 reads (Table 3), their presence remained unrecognized in the SNV frequency plots (Fig. 5).

## Discussion

In this study, using samples of commercially reared *A. domesticus,* we demonstrated the feasibility of a nanopore-based metagenomics sequencing approach for investigating the potential causative agents of diseased mass-reared insects. Genomic sequences of AdDV were successfully and reliably detected in a high number that reached up to 79.2% of total number of classified, high quality reads in symptomatic and deceased crickets, in comparison to non-symptomatic samples. In contrast, among the non-symptomatic samples, high number of viral reads were assigned to IIV6 for sample S13 and S16. IIV6 was previously reported in multiple metagenomics studies of cricket (*A. domesticus* and *G. bimaculatus*) as covert infection where the virus has no apparent impact on the host^47,48^. Overall, this finding strongly suggests that AdDV is most likely the causative agent of disease in this colony of *A. domesticus*. In combination with the developed post sequencing bioinformatics pipeline and the custom index of insect pathogen sequences, coupled with the NVIDIA Jetson AGX Xavier Development Kit as a central control unit, nanopore based metagenomics sequencing allows the diagnosis process of diseased insects and identification of potential entomopathogens within less than 24 h. Beyond a rapid and reliable diagnosis of AdDV, our approach additionally allowed the recovery of complete pathogen genomes and provided an in-depth insight into the genetic composition, genome-wide genetic variation and phylogenetic relationship of the pathogen to other AdDV isolates. While our study did not incorporate a direct comparison of metagenomics analysis using short reads sequencing techniques, several studies, particularly within the field of medical research have demonstrated the outcome from Illumina and nanopore-based metagenomics achieved similar rates of pathogen detection in clinical diagnosis^49,50^.

Metagenomics sequencing of *A. domesticus* samples revealed the presence of a vast diversity of bacteria assigned to phyla Proteobacteria, Bacteroidetes and Firmicutes. This finding is not surprising, as they have been reported in several studies investigating gut bacterial communities of insects, including *A. domesticus*^51–53^. In addition to that, the detection of bacteria from genera *Citrobacter*, *Fusobacterium* and *Klebsiella* corroborates earlier findings of the presence of these bacteria in the gastrointestinal tract of *A. domesticus*^54,55^. Overall, the result here suggest that the bacterial genera detected are from the common bacterial community of the house cricket. On the other hand, the majority of the unclassified reads were assigned to the genomes of *A. domesticus*, closely related insect species or other eukaryotes. This finding highlights the significance of a reference database on a target independent metagenomics screening approach^48^. Curated data of entomopathogens in the publicly available database such as NCBI remained scarce in comparison to medically important pathogens^48^, which undermines the potential application of metagenomics sequencing workflow in the diagnosis of entomopathogens. However, as a routine surveillance and portable diagnostic tool, this methodology has proven effective in rapidly identifying a diverse array of bacteria and viruses. Subsequently, it allows for the screening of potential entomopathogens within a relatively short timeframe.

The obtained AdDV sequences belonged to three different phylogenetic lines, namely clades A to C. Strikingly, the different clades displayed correlation to the vital status of diseased crickets, whereby AdDV detected in deceased crickets fell into the clades A or B (except S2), and AdDV from alive but symptomatic samples clustered in clade C. Among the five ORFs of AdDV, replication initiator protein (NS1) was found to be most conserved, with five non-synonymous substitutions found across the three clades. The helicase domain (position 424–526 aa) of NS1 remains intact for all the samples, with one amino acid substitution for clade A and C. In contrast, NS3 gene was found with the highest genetic variation (represented by the SNV density), which is in agreement with a previous study^13^. Limited information is available regarding the role of NS3 in AdDV. In contrast, in the example of Junonia coenia densovirus (JcDV) and Bombyx mori densovirus (BmDV), NS3 protein has been shown to play a crucial role in the replication of viral DNA^56,57^. The impact of these substitutions on virus’ pathogenicity remains unknown and warrants further investigation.

AdDV is mainly present in a covert state and there is no current information on how the virus can switch from a covert to an active state, causing virus outbreaks^58^. A study from Takacs, et al.^58^ demonstrated that density and temperature of rearing condition play a minimal role in AdDV viral abundance. Beyond abiotic factors, from our results, it could be hypothesized that different AdDV genotypes, displaying different pathogenicity of AdDV might play a role in the onset of symptoms, ultimately causing the death of insects. Similar observation is noted on the well-studied DWV (family *Iflaviridae*) of honey bees, associated with the ectoparasitic mite *V. destructor,* its biological and mechanical vector. Three different variants, DWV-A, DWV-B and DWV-C were reported, but several studies have demonstrated, using both molecular and microscopic evidences, that DWV-B is more virulent^59–61^. It was shown that different DWV variants are able to coexist within the same host. Additionally, the small genome size of AdDV allowed deciphering of individual AdDV genomes by PCR amplification and Sanger sequencing in previous studies^45,62^. However, these methods risk the loss of information about genetic variation, such as individual genotypes in single AdDV populations. By utilizing the advantage of long reads sequencing, which covers almost the entire virus genome, our study unraveled the presence of different genotypes of AdDV in individuals of *A. domesticus* based on SNV frequency.

In summary, our study demonstrates that metagenomics sequencing as developed here, has the potential to be a highly valuable tool for the routine surveillance and rapid diagnosis of entomopathogens within the expanding domain of mass-reared insects. We were not able to detect viral sequences of AdDV in apparently healthy samples, although covert or asymptomatic AdDV infection has been reported in *A. domesticus* colonies^47,63^. This might be due to an insufficient depth of sequencing run, that the viral titer level exceeded the limit of detection of nanopore-based whole metagenomics sequencing, or simply because the virus is not present in the healthy samples. Future studies on comparison of different detection methods, for instance quantitative PCR assays developed by Semberg, et al.^18^ will be informative and crucial to gauge the sensitivity of nanopore based diagnosis protocol^48^. Nevertheless, the capability of this approach to discern individual genotypes or a mix of genotypes in entomopathogens based on inherent SNV patterns not only expedites detection but also contributes to a deeper comprehension of the underlying pathogenic processes.

### Supplementary Information

Supplementary Information 1.

Supplementary Information 2.

Supplementary Information 3.

Supplementary Information 4.

Supplementary Information 5.

Supplementary Information 6.

## Data Availability

This data presented, including the assembled genomes (PP054196 to PP054217) in this study are uploaded and openly available at the National Centre of Biotechnology Information (NCBI) Sequence Read Archives (SRA) under the BioProject PRJNA996909.
