# Supplementary material for: Advancing pathogen surveillance by nanopore sequencing and genotype characterization of Acheta domesticus densovirus in mass-reared house crickets
Source: Sci Rep. 2024 Apr 12;14:8525. doi: 10.1038/s41598-024-58768-3 (PMC11014933; doi:10.1038/s41598-024-58768-3)
Supplement: Supplementary file 2 — Supplementary Information 2. [file 41598_2024_58768_MOESM2_ESM.pdf]

S2. Table: Accession Number of AdDV sequences used in this study

Accession Number	Sequences Name	Origin
NC_004290.1	AdDNV Ref	-
HQ827781.1	AdSw77	Swiss
KF015274.1	AdEu04	Germany
KF015275.1	AdEu06	Germany
KF015276.1	AdEu07	Germany
KF015277.1	AdEu09	Germany
KF015278.1	AdNA09	North America
KF015279.1	AdJP12	Japan
PP054196	AdDV S1	This study
PP054197	AdDV S2	This study
PP054198	AdDV S2.1	This study
PP054199	AdDV S2.2	This study
PP054200	AdDV S3	This study
PP054201	AdDV S4	This study
PP054202	AdDV S5	This study
PP054203	AdDV S6	This study
PP054204	AdDV S7	This study
PP054205	AdDV S7.1	This study
PP054206	AdDV S7.2	This study
PP054207	AdDV S8	This study
PP054208	AdDV S8.1	This study
PP054209	AdDV S8.2	This study
PP054210	AdDV S9	This study
PP054211	AdDV S9.1	This study
PP054212	AdDV S10	This study
PP054213	AdDV S11	This study
PP054214	AdDV S11.1	This study
PP054215	AdDV S11.2	This study
PP054216	AdDV S12	This study
PP054217	AdDV S12.1	This study
