# Supplementary material for: Advancing pathogen surveillance by nanopore sequencing and genotype characterization of Acheta domesticus densovirus in mass-reared house crickets
Source: Sci Rep. 2024 Apr 12;14:8525. doi: 10.1038/s41598-024-58768-3 (PMC11014933; doi:10.1038/s41598-024-58768-3)

S4. Figure Sankey Plots of S1 to S18

Sample S1

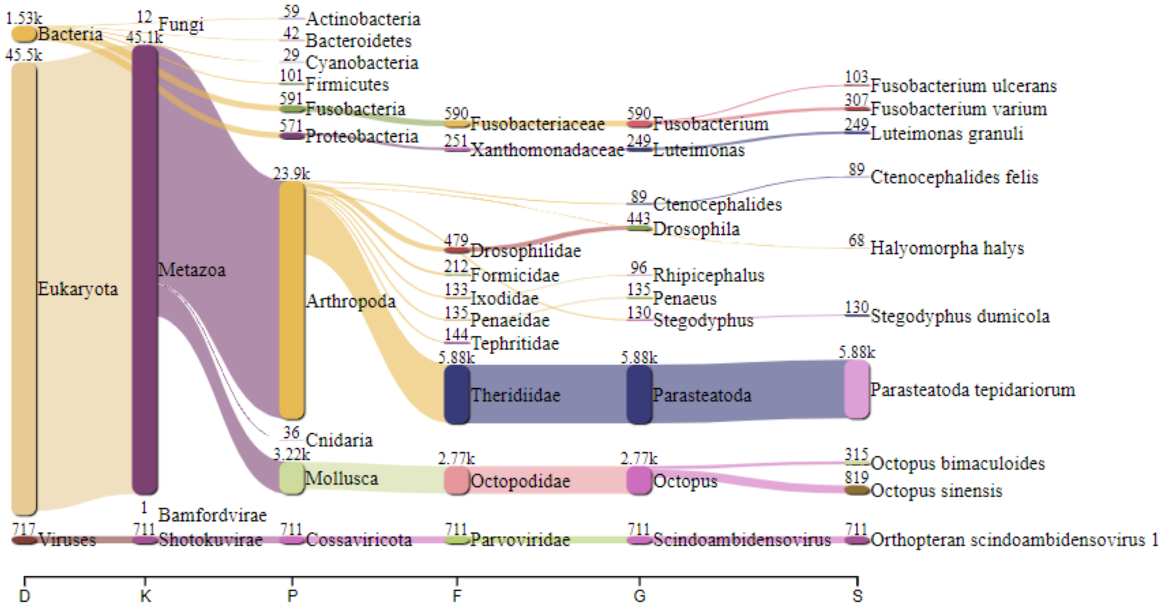

Sample S2

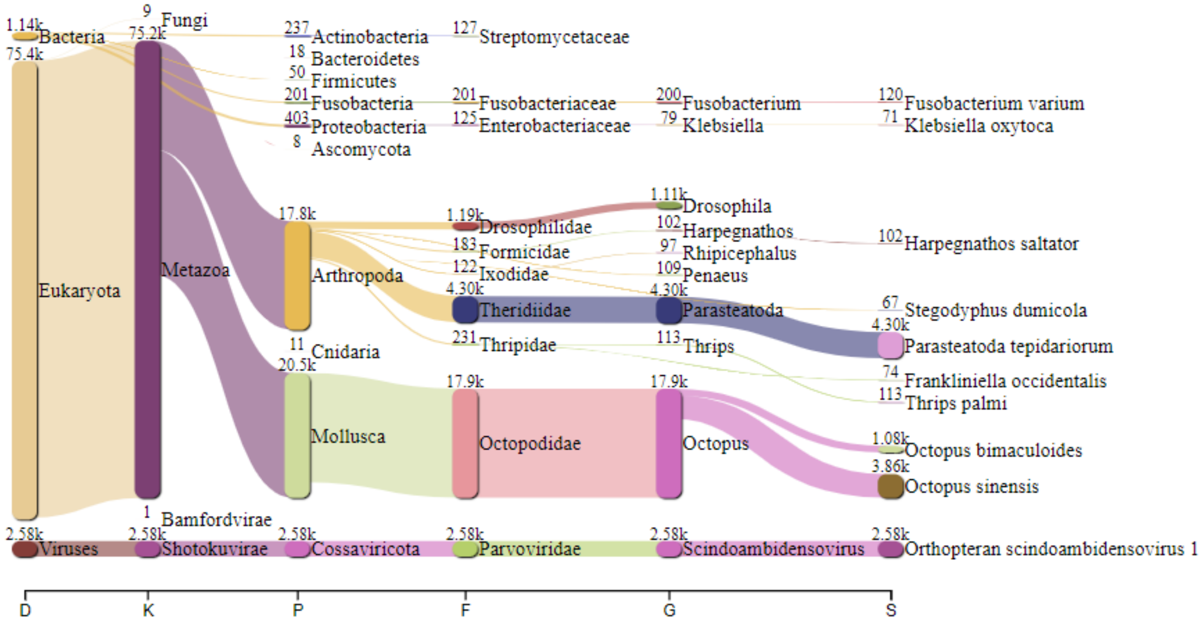

Sample S3

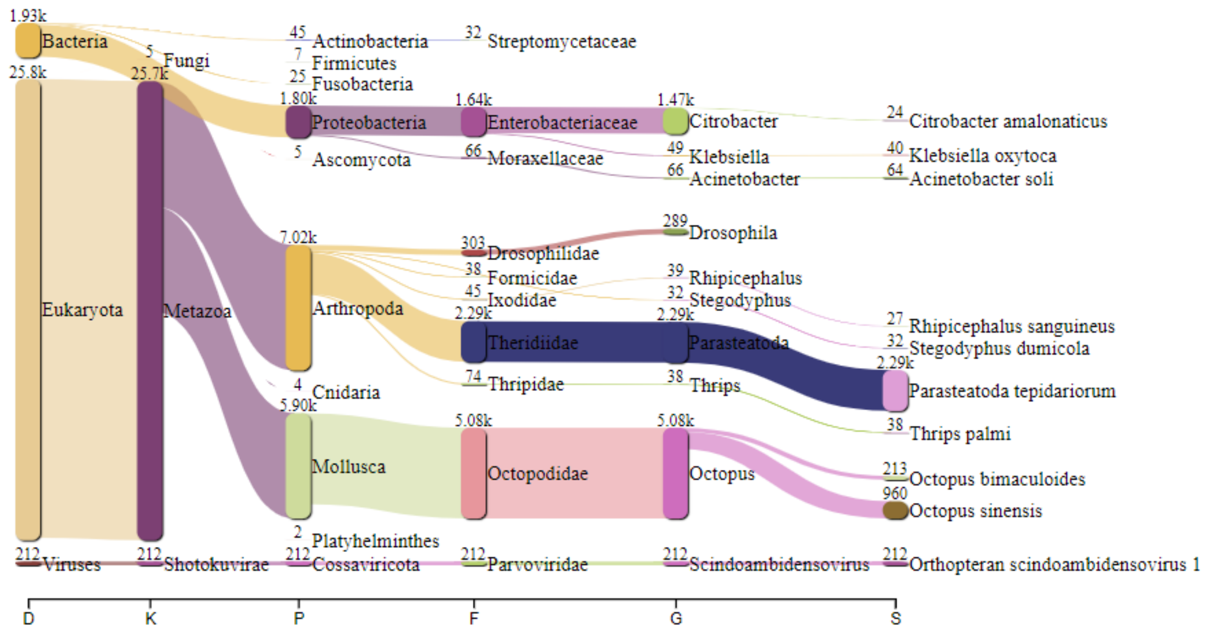

Sample S4

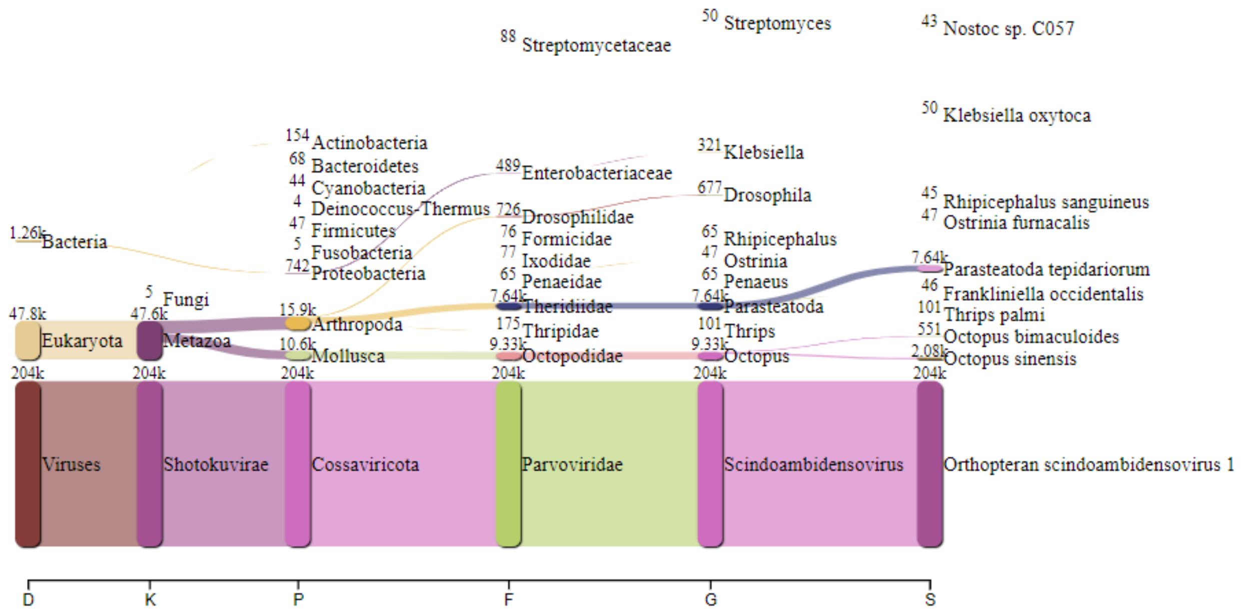

Sample S5

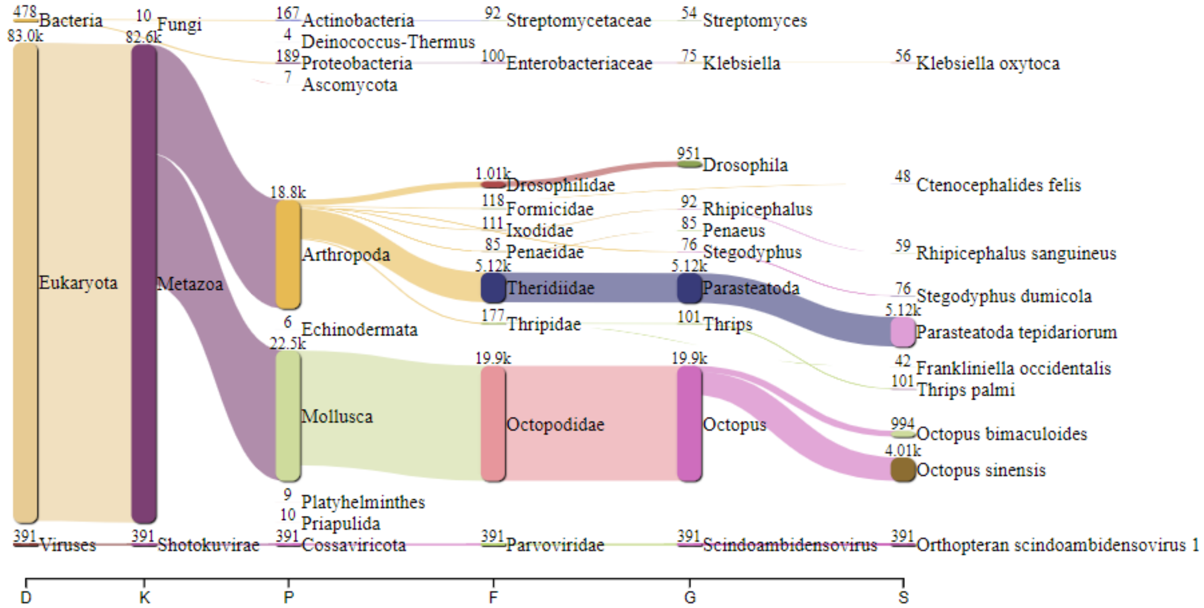

Sample S6

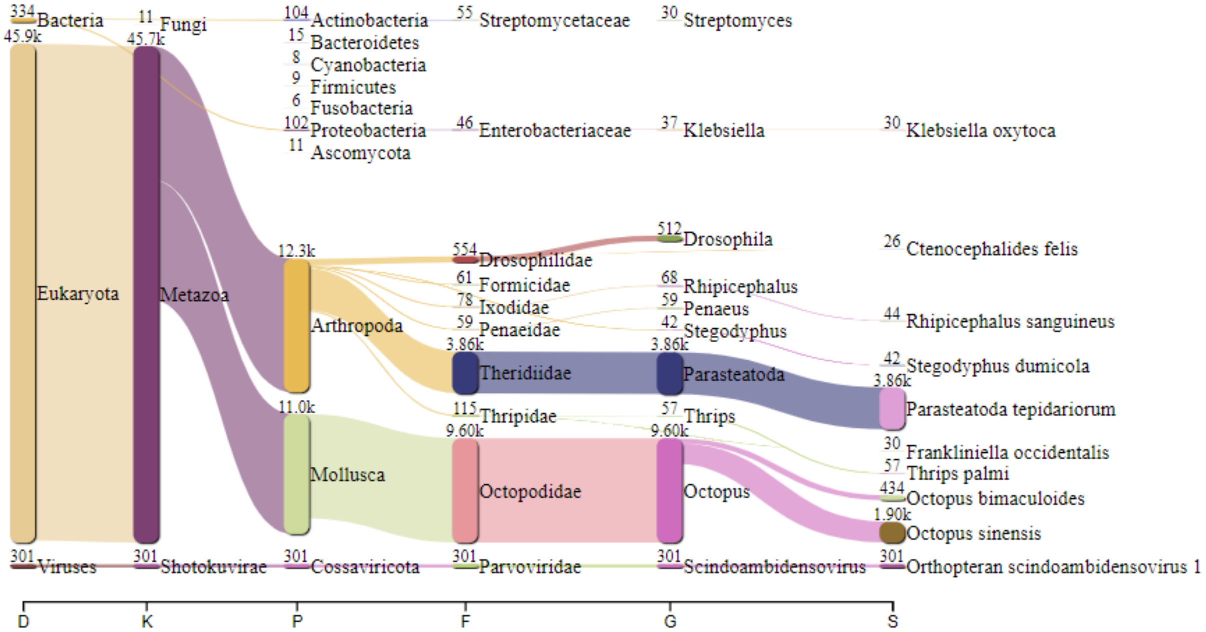

Sample S7

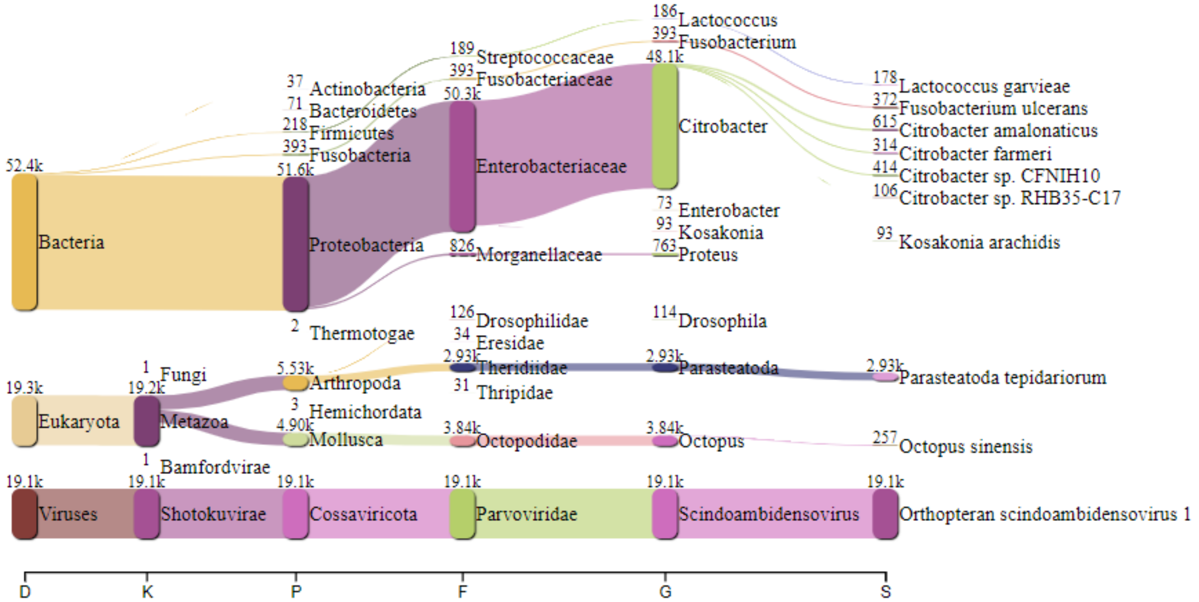

Sample S8

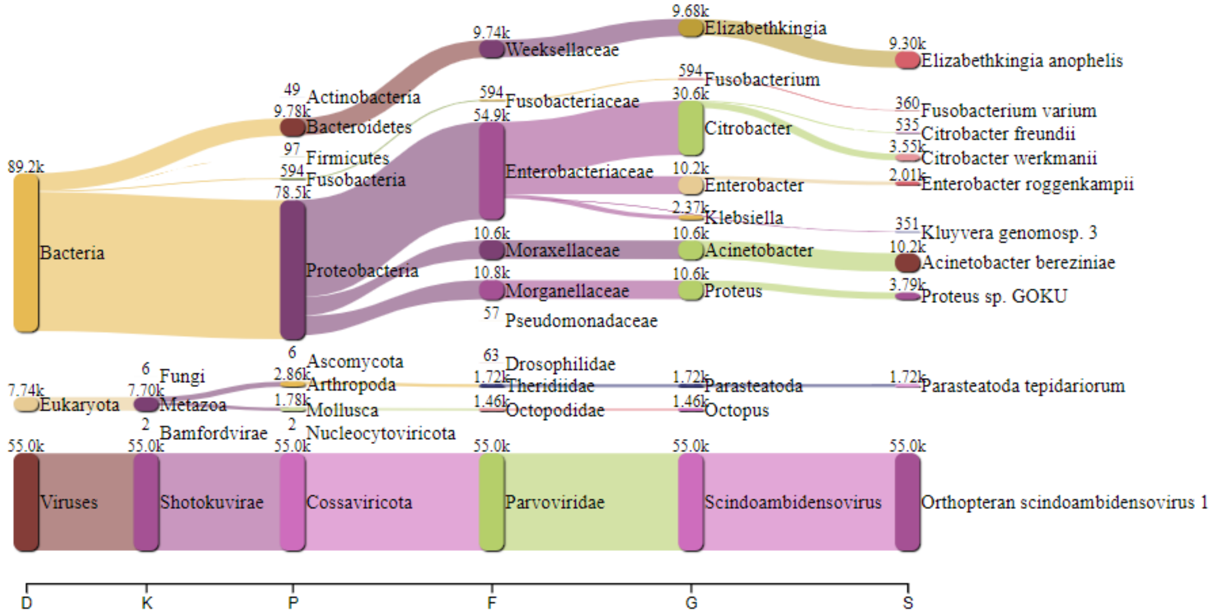

Sample S9

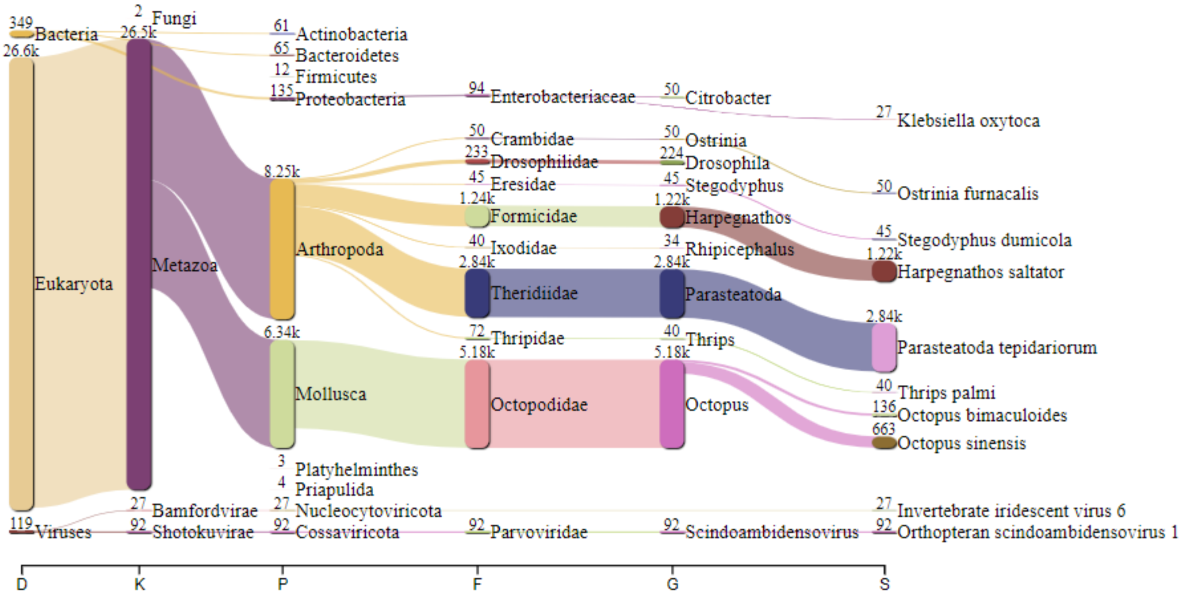

Sample S10

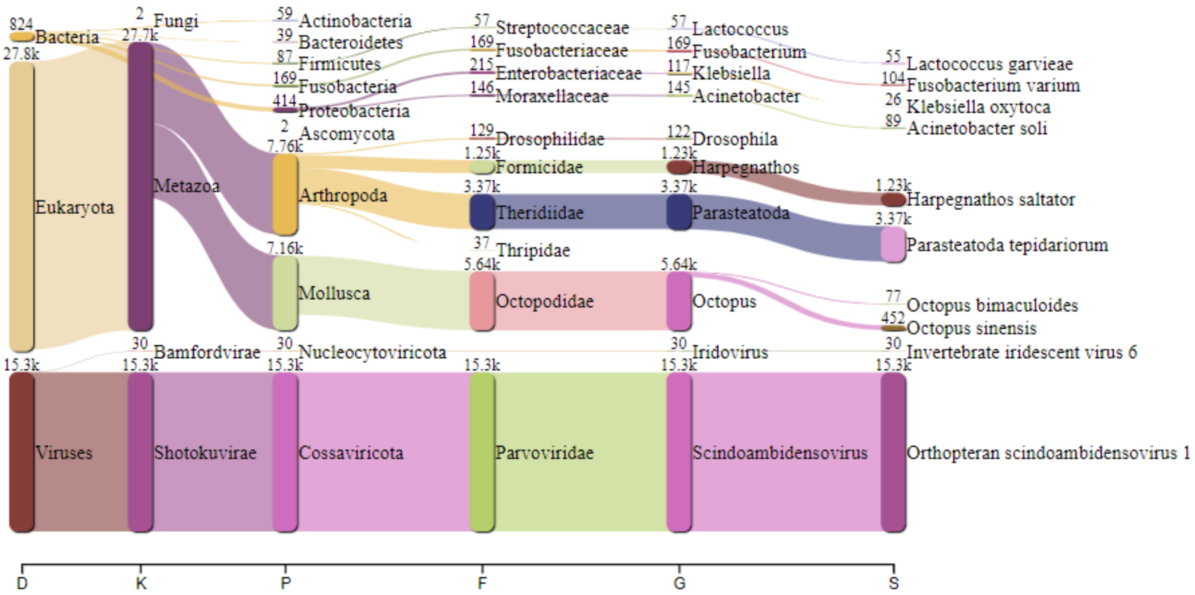

Sample S11

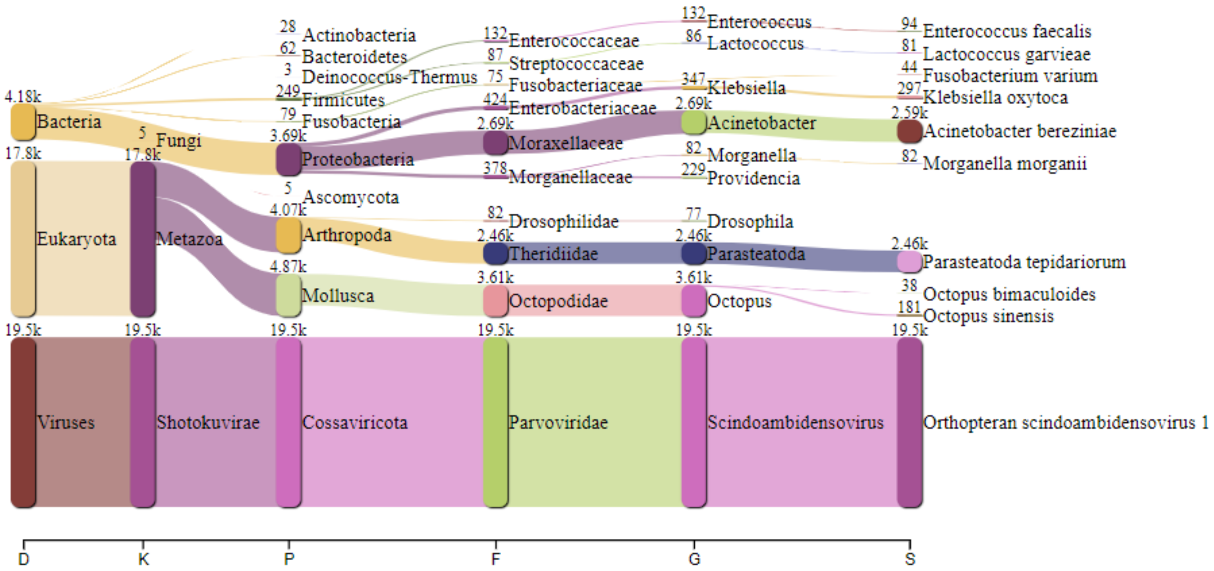

Sample S12

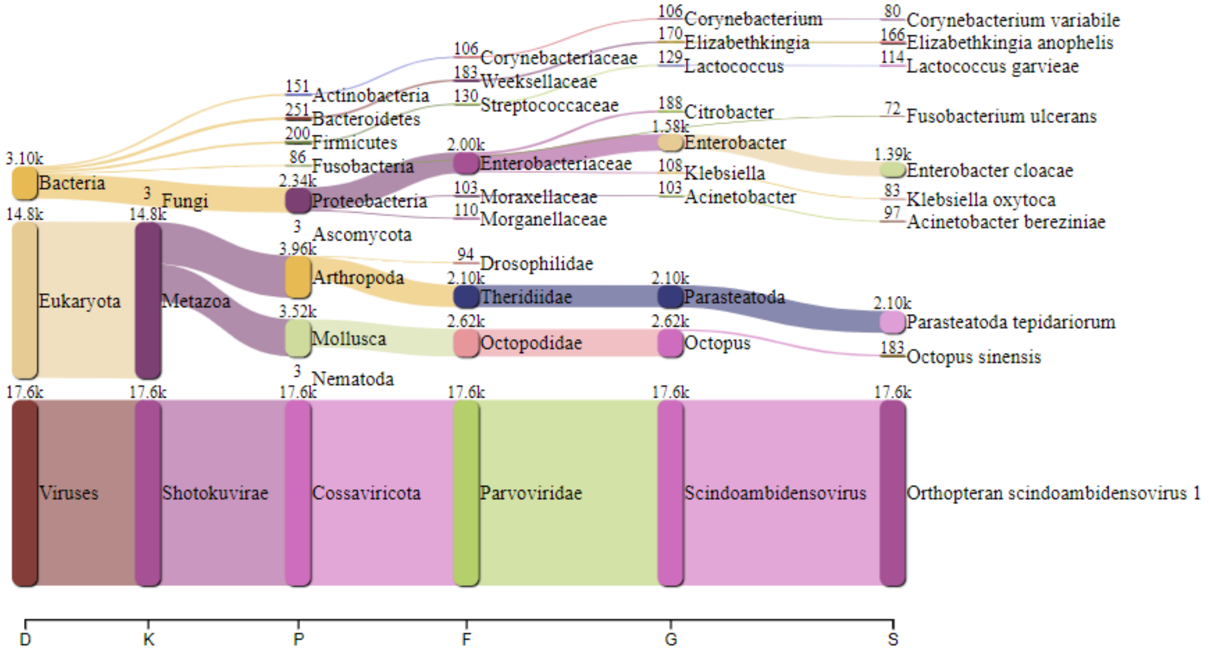

Sample S13

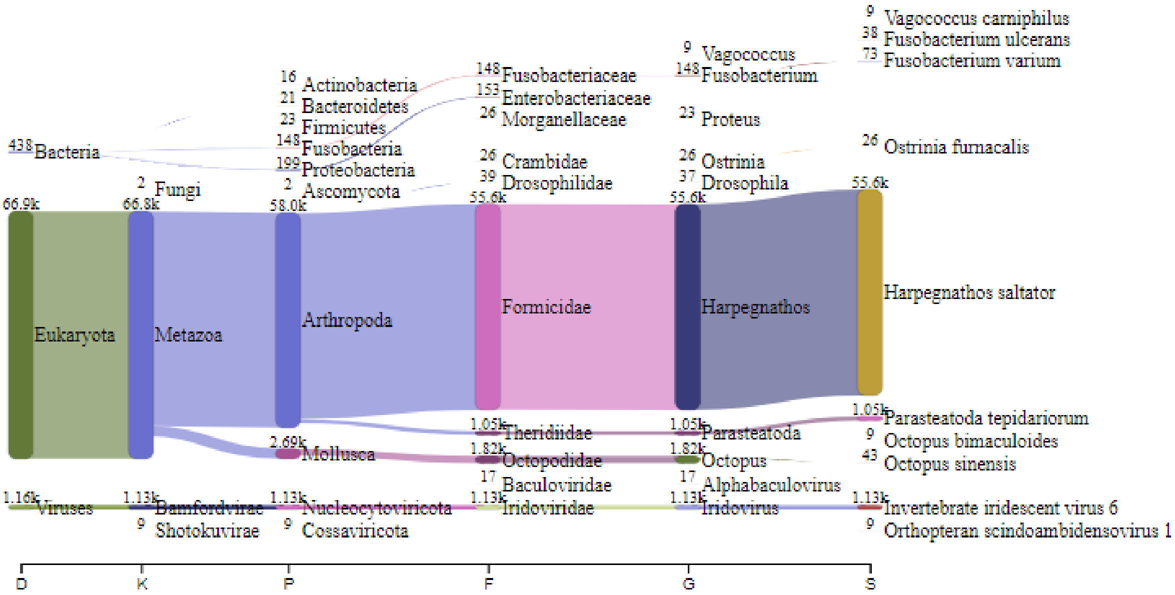

Sample S14

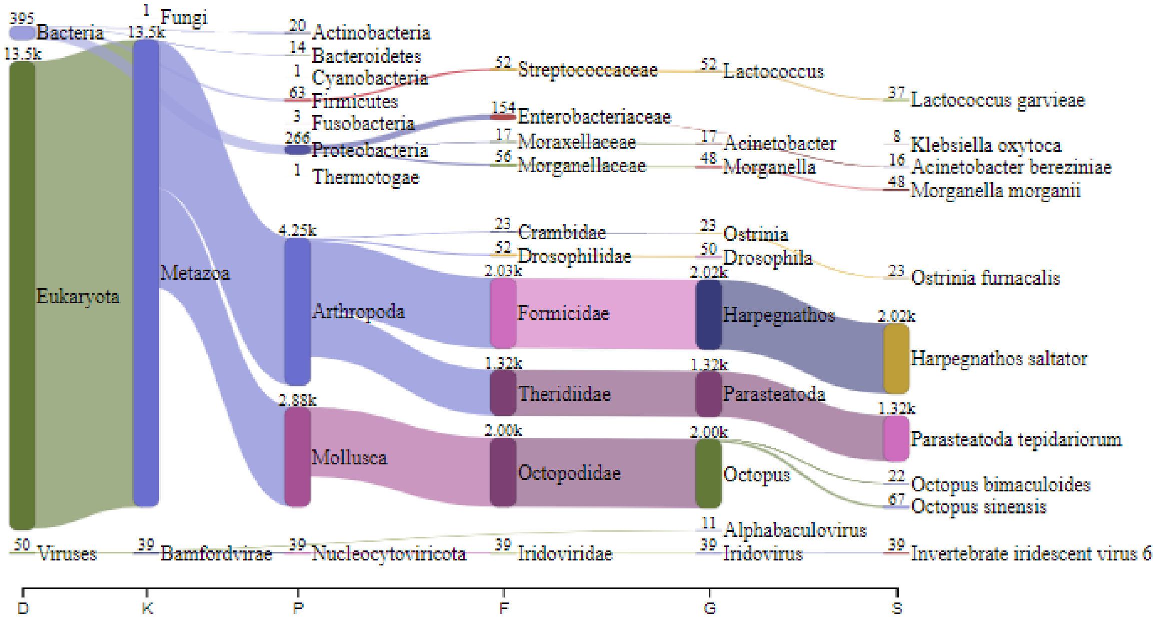

Sample S15

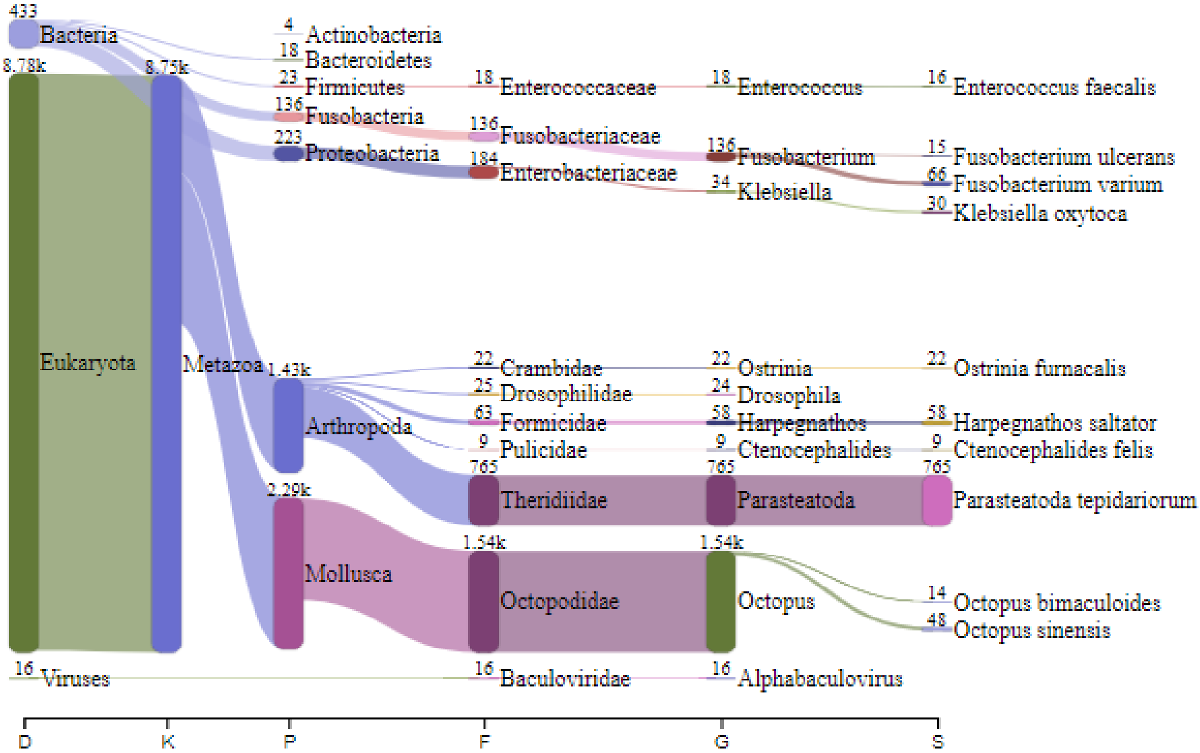

Sample S16

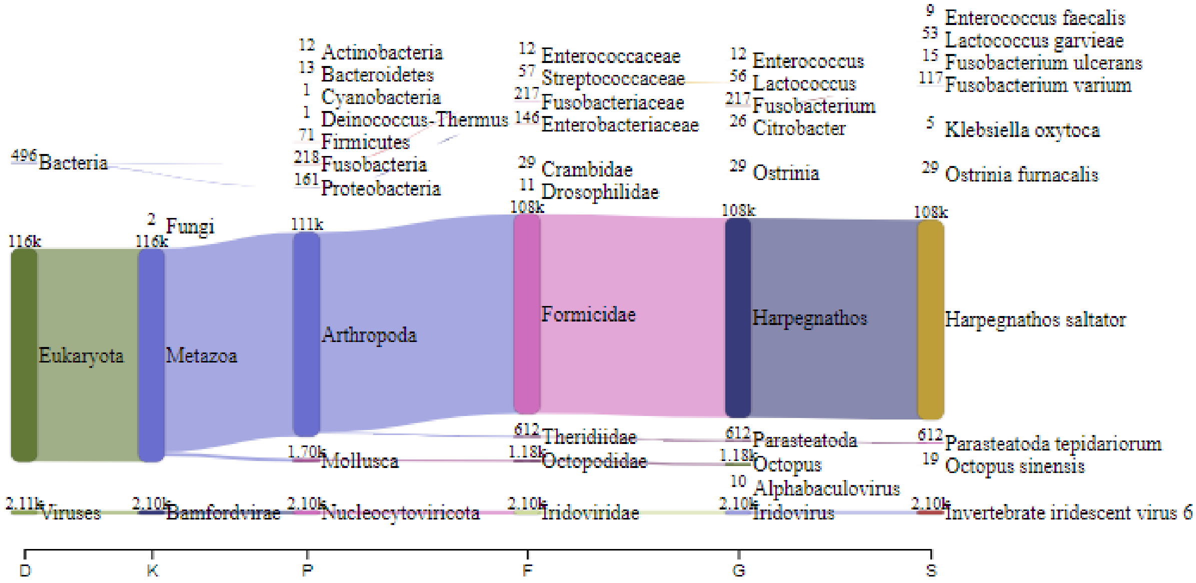

Sample S17

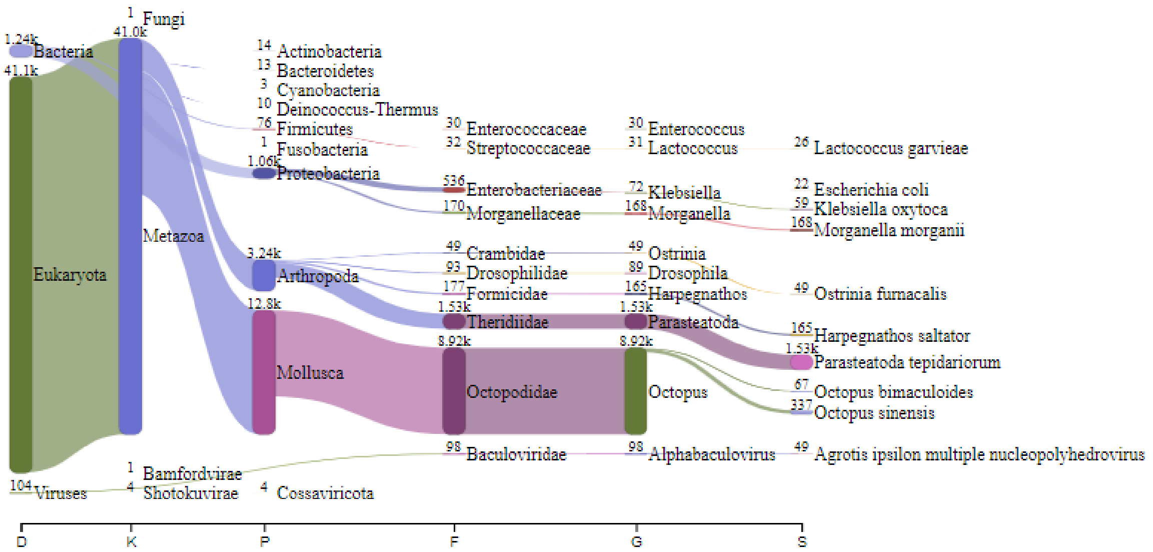

Sample S18

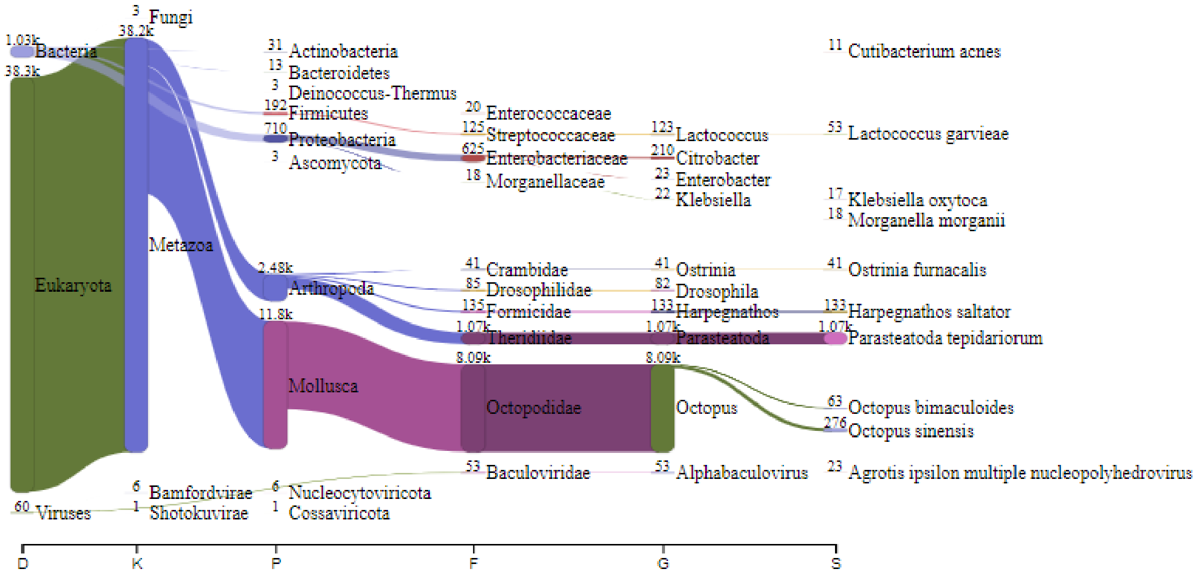

Supplement: Supplementary file 4 — Supplementary Information 4. [file 41598_2024_58768_MOESM4_ESM.pdf]
