# Supplementary material for: Advancing pathogen surveillance by nanopore sequencing and genotype characterization of Acheta domesticus densovirus in mass-reared house crickets
Source: Sci Rep. 2024 Apr 12;14:8525. doi: 10.1038/s41598-024-58768-3 (PMC11014933; doi:10.1038/s41598-024-58768-3)
Supplement: Supplementary file 6 — Supplementary Information 6. [file 41598_2024_58768_MOESM6_ESM.pdf]

Advancing Pathogen Surveillance by Nanopore Sequencing and Genotype Characterization of Acheta domesticus densovirus in Mass-Reared House Crickets

Fang Shiang Lim[1, 2], Joel González-Cabrera[2], Jens Keilwagen[3], Regina G. Kleespies[1], Johannes A. Jehle[1], Jörg T. Wennmann[1*]

Supporting Information Captions

S1. Appendix Library inspection of genome sequences presence in custom built RFGINV_JKI_ID 1.0 index

S2. Table Accession Number of AdDV sequences used in this study

S3. Table BLASTn result of unclassified contigs from S1 to S12

S4. Figure Sankey Plots of S1 to S18

S5. Appendix Full taxonomic assignment list
